# External validation of PREDICT Breast v3.1 for overall survival in international cohorts, including young and invasive lobular subgroups

**DOI:** 10.1007/s10549-026-07958-w

**Published:** 2026-04-17

**Authors:** Elfi M. Verheul, Frank Doornkamp, Iurii Petrov, Sabine Siesling, Hester F. Lingsma, Linetta B. Koppert, Lara W. A. Vreven, Adri C. Voogd, Maria Margarete Karsten, Lea Doppelbauer, Pimrapat Gebert, Narsis Kiani, Simona Borstnar, Paul D. P. Pharoah, Elham Hedayati, Ewout W. Steyerberg, David van Klaveren

**Affiliations:** 1 Center for Medical Decision Making, Department of Public Health, Erasmus University Medical Center, Rotterdam, The Netherlands; 2 Dutch Institute for Clinical Auditing, Leiden, The Netherlands; 3 Biomedical Data Sciences, Leiden University Medical Center, Leiden, The Netherlands; 4 Microbiology, Tumour- and Cell Biology Department, Karolinska Institutet, Stockholm, Sweden; 5 Cancer Centrum Karolinska (CCK), Solna, Sweden; 6 Department of Research, Netherlands Comprehensive Cancer Organization (IKNL), Utrecht, The Netherlands; 7 Department of Health Technology and Services Research, Technical Medical Centre, University of Twente, Enschede, The Netherlands; 8 Department of Surgery, Erasmus MC Cancer Institute, Rotterdam, The Netherlands; 9 Department of Epidemiology, Maastricht University, Maastricht, The Netherlands; 10 Department of Gynecology with Breast Center, Charité - Universitätsmedizin Berlin, Berlin, Germany; 11 Berlin Institute of Health at Charité -Universitätsmedizin Berlin, Berlin, Germany; 12 Institute of Biometry and Clinical Epidemiology, Charité - Universitätsmedizin Berlin, Berlin, Germany; 13 Department of Oncology-Pathology, Center for Molecular Medicine, Karolinska Institutet, Solna, Sweden; 14 Algorithmic Dynamics Lab, Karolinska Institutet, Solna, Sweden; 15 Department of Medical Oncology, Institute of Oncology Ljubljana, Ljubljana, Slovenia; 16 Department of Computational Biomedicine, Cedars-Sinai Medical Center, Los Angeles, CA USA; 17 Breast Cancer Centre, Cancer Theme, Karolinska University Hospital, Karolinska CCC, Stockholm, Sweden; 18 Julius Center, University Medical Center Utrecht, Utrecht, The Netherlands

**Keywords:** Breast cancer, PREDICT breast, External validation, Prediction model, Overall survival

## Abstract

**Purpose:**

PREDICT Breast is an online tool that provides survival predictions for patients with early-stage breast cancer, for different treatments after surgery. External validation is essential to assess model performance across populations and healthcare settings. We aimed to externally validate PREDICT using clinical practice data from the Netherlands, Sweden, and Slovenia.

**Methods:**

We validated PREDICT in national populations (*Netherlands, N* = *221,636; Sweden, N* = *84,928)* and in two specific subgroups: patients with invasive lobular breast cancer (ILC) *(Netherlands, N* = *26,834; Sweden, N* = *10,563; Slovenia, N* = *341)* and patients aged ≤ 40 years *(Netherlands, N* = *9995; Sweden, N* = *2694)*. We assessed discrimination with the 10-year area under the curve (AUC) and calibration of 10-year mortality predictions through calibration plots, intercepts and slopes.

**Results:**

PREDICT v3.1 discriminated well in the national populations (Netherlands AUC 0.75, 95% CI 0.75–0.76; Sweden 0.75, 95% CI 0.75–0.76), with similar discrimination in ILC patients (Netherlands 0.76, 95% CI 0.74–0.76; Sweden 0.75, 95% CI 0.73–0.77; Slovenia 0.78, 95% CI 0.71–0.83). Calibration showed slight underestimation of mortality risk in the Netherlands (intercept 0.13; slope 1.01), and was near perfect in the Swedish population (intercept 0.04; slope 1.05). Amongst ILC patients, we observed some underestimation of mortality (Netherlands intercept 0.20; Sweden intercept 0.10; Slovenia intercept 0.02). In young patients, miscalibration was observed (Netherlands, intercept 0.21, slope 0.79; Sweden, intercept 0.08, slope 0.85).

**Conclusion:**

PREDICT v3.1 is generally well calibrated and suitable for clinical use in the evaluated European populations. Efforts to improve PREDICT should focus on more accurate predictions for younger patients.

**Supplementary Information:**

The online version contains supplementary material available at 10.1007/s10549-026-07958-w.

## Introduction

Shared decision-making (SDM) and personalised care are increasingly recognised as key components of modern cancer care. Prediction models can support SDM [1], and several have been developed for breast cancer (BC) [2, 3], with PREDICT being widely used in clinical practice [4].

External validation of prediction models is essential to assess model performance across diverse populations and healthcare settings beyond those used for their development. This is important as patient characteristics, clinical protocols, and treatment practices can vary substantially, both between and within countries, and may also change over time [5]. Validation focuses on discrimination (distinguishing between patients with different outcomes) and calibration (agreement between predicted and observed outcomes) [6]. PREDICT is routinely updated, with the most recent version (3.1, released in 2024) introducing several improvements, including a refitted model based on more recent data (2000–2017), and accounting for both the beneficial effect of radiotherapy on breast cancer mortality and the harmful effects of chemotherapy and radiotherapy on other causes of death[7]. This version has not yet been externally validated in European cohorts beyond the UK.

Histology is not included in v3.1, as it did not demonstrate independent prognostic value during model development, although clinically relevant differences remain between invasive ductal carcinoma (IDC) and invasive lobular carcinoma (ILC). Patients with ILC are typically older, present with more advanced tumours, and show higher ER and lower HER2 expression, with mastectomy more common than breast-conserving surgery [8–12]. More importantly, ER-positive ILC is associated with a poorer long-term disease-free and overall survival [9, 11] and adjuvant chemotherapy confers less benefit in patients with ILC, although selected patients may still benefit [13, 14]. Additionally, prediction models often perform less consistently in young patients [3], including reports of underestimation of mortality by PREDICT in young, node-negative cases [15]. Although women aged 40 years or younger represent a relatively small subgroup, their long life expectancy makes accurate risk prediction essential for guiding treatment. These gaps underscore the need for dedicated validation in both young patients and those with ILC.

We aim to externally validate PREDICT v3.1 using multiple datasets from European cancer registries. We evaluate its performance in the Dutch and Swedish national cancer populations and further assess its validity in clinical subgroups where its use may be uncertain: patients with ILC (including Slovenia) and patients aged 40 years and younger.

## Materials and methods

### Data and study population

As part of the 4D project, which aims to enhance data-driven decision-making in oncology [16], this study includes datasets from multiple countries:

- The Netherlands Cancer Registry (NCR; Netherlands) includes all breast cancer patients diagnosed between.
- BcBaSe 3.0 (Sweden) contains research database based on the Swedish National Quality Breast Cancer Register, covering all breast cancer patients in Sweden from.
- ILC Database, Institute of Oncology, Ljubljana (Slovenia) contains all invasive lobular carcinoma (ILC) patients diagnosed between 2003 and 2008 at the Institute of Oncology, Ljubljana.

The inclusion criteria are comparable to those of the PREDICT Breast development set [7, 17]. We selected female patients aged between 25 and 85 years with invasive breast cancer, without distant metastasis. Patients who did not undergo surgery, received neoadjuvant chemotherapy, had tumours larger than 200 mm, or had more than 20 positive lymph nodes were excluded.

For the subgroups, patients with ILC were defined using ICD-O-3.2 code 8520 [18]. Patients aged 40 years or younger were defined based on age at the time of diagnosis (biopsy). Given the hypothesised miscalibration in this subgroup, we additionally evaluated calibration across other age groups to assess whether the miscalibration is specific to younger patients.

#### Predictors and treatment characteristics

The predictors used in the PREDICT algorithm are age at diagnosis, smoking status, ER status, PR status, HER2/ERRB2 status, Ki-67 status (positive defined as more than 10%), invasive tumour size, tumour grade, method of detection (screening or symptomatic), and positive lymph nodes, including micrometastases only when the number of positive nodes is one. Whilst postmenopausal status appears in the online tool to allow selection of bisphosphonate therapy, it is not included in the prognostic model. All predictors were selected from the different databases based on their availability. Since smoking status was not available in any database, we assumed a fixed prevalence of 15% based on a prior Dutch study [19].

Treatment characteristics included in the v3.1 version of the tool are radiotherapy, hormone therapy, chemotherapy, trastuzumab, and bisphosphonates. Chemotherapy regimens were categorised as ‘standard dose’, referring to anthracycline-based, second-generation regimens, such as fluorouracil, epirubicin, cyclophosphamide (FEC), and ‘high-dose’, referring to third-generation regimens, either high cumulative anthracycline or anthracycline combined with a taxane (e.g. paclitaxel, docetaxel) [20]. If the type of chemotherapy was unknown, patients were assumed to have received high-dose chemotherapy, as this represents the most common practice [21, 22]. Furthermore, we assumed that if treatment was given, it was also completed. For radiotherapy, 2 Grey was assigned for left-sided and 0 Grey for right-sided treatment in the Netherlands and Sweden, consistent with the tool’s assumptions. For Slovenia, where laterality was missing, 1 Grey was assigned to all patients who underwent radiotherapy. Hormone therapy was assumed to last 5 years in line with guidelines [23]. As trastuzumab data were unavailable in the Slovenian dataset, we assumed that all HER2-positive tumours received this treatment [24]. Bisphosphonate treatment was set to zero in all databases due to lack of information, which has previously been shown not to significantly impact the results (Table S1) [25].

#### Outcomes

We focussed on 10-year overall survival (OS), considering death from any cause without differentiating underlying causes of mortality. Additional analyses of 5-year outcomes are provided in the supplementary material.

#### Statistical analysis

Descriptive statistics were reported for each predictor, treatment characteristic, and outcome used in the PREDICT tool for the different cohorts, with means and standard deviations (SD) for continuous variables and frequencies and percentages for categorical variables.

The PREDICT algorithm v3.1 was obtained from the developer (P.D.P. Pharoah [7]) and used in RStudio to estimate survival probabilities. Variables with missing data that could be selected as ‘unknown’ by the PREDICT algorithm were treated as such (PR status, HER2 status, Ki-67 status, and mode of detection). Any missing data in the input variables, without the option of ‘unknown’ in the algorithm (age, ER status, tumour size, tumour grade, positive nodes), were assumed missing at random and imputed using MICE (20 imputed data sets). We used the PredictionTools Package [26], optimised for estimating statistical metrics for survival predictions with imputed data sets, to make calibration plots and estimate discriminative performance metrics.

The discriminative ability of the prediction models was assessed with the AUC (time-dependent area under the ROC curve; AUCt). We chose this AUCt over the C-index, since PREDICT provides estimates on 5- and 10-year survival probabilities rather than survival times [27]. We will hereafter refer to it simply as AUC. Model calibration was visually assessed with calibration plots and numerically assessed with calibration intercepts and slopes. Calibration plots visualise the agreement between predicted and observed outcomes (shown for 10-year mortality, the complement of predicted OS calculated as 1—OS). Expected survival is plotted on the horizontal axis, and observed survival on the vertical axis. Systematic deviations from the 45-degree line indicate miscalibration and are summarised with a calibration intercept and slope. A calibration intercept below 0 indicates systematic overestimation of risks (predicted risks are too high), whilst an intercept above 0 indicates underestimation. A slope less than 1 reflects too much spread in predictions, whereas a slope greater than 1 suggests too little. All performance measures were evaluated for the national population (full dataset), as well as for individual sub-cohorts (by country).

We reported the model validations following the TRIPOD checklist (Table S2). For all analyses, we used R statistical software version 4.4.2.

## Results

### Patient-, tumour-, and treatment characteristics

The national study population consisted of 221,636 Dutch and 84,928 Swedish patients (Table 1; Fig. S1A, B, C). Subgroups included patients with invasive lobular BC (ILC; Netherlands, *N* = 26,834; Sweden, *N* = 10,563; Slovenia, *N* = 341) and those aged 40 years or younger (Netherlands: *N* = 9,995; Sweden: *N* = 2,694).

**Table 1:** Predictors and treatment characteristics included in PREDICT Breast v3.1 for the validation cohorts from the Netherlands and Slovenia

	Netherlands (inclusion: 2003–2022)			Sweden (inclusion: 2007–2021)			Slovenia (inclusion: 2003–2008)
	Population*N* = 221,636	ILC *N* = 26,834	≤ 40 years *N* = 9996	Population *N* = 84,928	ILC *N* = 10,563	≤ 40 years *N* = 2694	ILC *N* = 341
Age at diagnosis (years)	61 (12)	63 (11)	36 (4)	62 (12)	65 (11)	36 (3)	62 (12) *1*
Missing							
Smoker	NA*	NA	NA	NA	NA	NA	NA
Missing							
Postmenopausal	NA	NA	NA	66,286 (80%)	8,846 (86%)	68 (3%)	248 (73%)
Missing				2319	299	43	1
ER status, positive	186,958 (87%)	25,595 (97%)	6737 (70%)	68,822 (88%)	10,363 (98%)	1766 (71%)	328 (96%)
Missing	6144	547	348	6406	33	196	
PR status, positive	150,936 (71%)	19,914 (77%)	5915 (62%)	58,095 (74%)	8479 (81%)	1559 (63%)	277 (81%)
Missing	9341	955	469	6498	40	200	
Her2/ERRB2 status, positive	20,511 (10%)	771 (3%)	1719 (20%)	9118 (12%)	437 (4%)	566 (23%)	19 (6%)
Missing	24,876	2717	1374	9204	366	262	2
Ki-67 status, positive	NA	NA	NA	40,240 (76%)	4738 (65%)	1410 (91%)	81 (24%)
Missing				32,275	3278	1138	
Invasive tumour size (mm)	18 (13)	25 (19)	20 (13)	18 (15)	26 (20)	20 (17)	25 (20)
Missing	8589	1159	485				3
Tumour grade	1 = 55,592 (26%)	1 = 4101 (16%)	1 = 1201 (13%)	1 = 16,670 (20%)	1 = 744 (7%)	1 = 223 (9%)	1 = 51 (15%)
	2 = 102,603 (48%)	2 = 18,936 (76%)	2 = 3417 (36%)	2 = 41,187 (50%)	2 = 8888 (85%)	2 = 847 (33%)	2 = 254 (74%)
	3 = 54,202 (26%)	3 = 2062 (8%)	3 = 4932 (52%)	3 = 23,928 (30%)	3 = 821 (8%)	3 = 1461 (58%)	3 = 36 (11%)
Missing	9239	1735	446	3143	110	163	
Detected by screening	59,665 (42%)	7009 (39%)	37 (< 1%)	46,776 (55%)	5456 (52%)	87 (3%)	NA
Missing	80,385	8910	4714	258	27		
Positive nodes, yes	66,927 (31%)	8842 (34%)	4101 (41%)	22,952 (27%)	3275 (31%)	1006 (37%)	132 (39%)
Missing	4544	474	65				
Micrometastases only	NA	NA	NA	5046 (6%)	648 (6%)	215 (8%)	30 (9%)
Missing							
Radiotherapy	151,169 (68%)	16,975 (63%)	6265 (63%)	49,626 (72%)	6263 (71%)	1381 (67%)	150 (44%)
Missing				16,183	1769	642	
Adjuvant hormonal therapy	111,982 (51%)	18,192 (68%)	5295 (53%)	50,283 (73%)	8145(93%)	1327 (65%)	322 (94%)
Missing				16,191	1770	638	
Adjuvant trastuzumab	12,600 (6%)	437 (2%)	1469 (15%)	5977 (7%)	262 (3%)	392 (15%)	1 (< 1%)
Missing							
Adjuvant chemotherapy**	No = 155,787 (70%)	No = 20,078 (75%)	No = 2477 (25%)	22,869 (33%)	2404(27%)	1559 (76%)	No = 263 (77%)
	Standard dose = 9,136 (4.1%)	Standard dose = 955 (4%)	Standard dose = 1060 (11%)				Standard dose = 66 (20%)
	High dose = 41,799 (19%)	High dose = 4,331 (16%)	High dose = 4488 (45%)				High dose = 12 (4%)
	Type unknown = 14,914 (7%)	Type unknown = 1,470 (5%)	Type unknown = 1971 (20%)				
Missing				16,203	1771	640	
Survival (5y)***	200,990 (91%)	24,342 (91%)	9344 (93%)	79,187 (93%)	9804 (93%)	2539 (94%)	303 (89%)
Survival (10y)	181,152 (82%)	21,630 (81%)	8817 (88%)	74,956 (88%)	9140 (87%)	3313 (91%)	253 (74%)

Values are *n* (%), unless for age and invasive tumour size, which are presented as mean (standard deviation)

*ILC* invasive lobular breast cancer patients, *NA* not present in the dataset

*81% missing, excluded from predictions

**Detailed information about the type of adjuvant chemotherapy was not available for the Swedish cohort

***Results of 5-year analysis are included in the supplementary material

For the national populations, most patient and tumour characteristics were comparable. Differences were observed in screen detection rates, which were higher in Sweden than in the Netherlands (55% vs 42%). More patients received hormone therapy in Sweden (73% vs 51%), but no differences were observed in the proportion of patients with ER-positive disease (88%, 87%, respectively). In terms of outcome, OS after 10 years was better in Sweden, with 74,956 out of 84,928 patients surviving (88%), compared to 181,152 out of 221,636 patients (82%) in the Netherlands. Almost all patients with ILC had ER-positive tumours (Netherlands: 97%; Sweden 98%; Slovenia 96%), resulting in a high proportion receiving hormone therapy. Tumours in young patients (≤ 40 years) were less likely to be ER-positive (Netherlands 70% vs 87%; Sweden 71% vs 88%), more likely to be HER2-positive (Netherlands 20% vs 10%, Sweden 23% vs 12%) and more often poorly differentiated (grade 3 Netherlands 52% vs 26%, Sweden 58% vs 30%) compared with the overall national populations, which is consistent with the higher proportion of young patients receiving adjuvant trastuzumab (anti-HER2 therapy) and adjuvant chemotherapy.

### Validation of PREDICT Breast v3.1

**Fig. 1:**
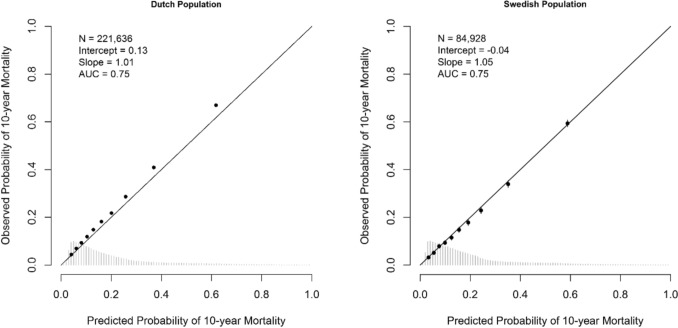
Calibration plots for the Dutch (left) and Swedish (right) populations, showing 10-year overall mortality predictions using PREDICT Breast v3.1. *N*, number of patients included in the external validation set; AUC, area under the curve at 10 years. The intercept and slope (ideally 0 and 1, respectively) are measures used to evaluate calibration. The *Y*-axis represents the observed 10-year mortality, and the *X*-axis represents the predicted 10-year mortality. Each dot indicates the average for a decile of predicted probabilities, with accompanying 95% confidence intervals (95% CI; due to the large sample size, this is negligible in this figure)

External validation in the two national populations showed good discrimination at 10 years, with an AUC of 0.75 (95% CI 0.75–0.76) in the Netherlands and 0.75 (95% CI 0.75–0.76) in Sweden (Fig. 1). Mortality risk is slightly underestimated in the Netherlands (intercept 0.13; slope 1.01) and shows near-perfect calibration in Sweden (intercept 0.04; slope 1.05). When stratifying by oestrogen receptor (ER) status, underestimation was more pronounced among patients with ER-positive tumours in the Netherlands (ER^+^ intercept 0.18, slope 1.03; ER^−^ intercept − 0.05, slope 0.97; Fig. S2A), whereas in Sweden patients with ER-positive tumours contributed to the near-perfect calibration (ER^+^ intercept − 0.02, slope 1.06; ER^−^ intercept − 0.11, slope 0.96; Fig. S2B). Calibration plots for 5-year overall mortality showed similar results (Fig. S3).

**Fig. 2:**
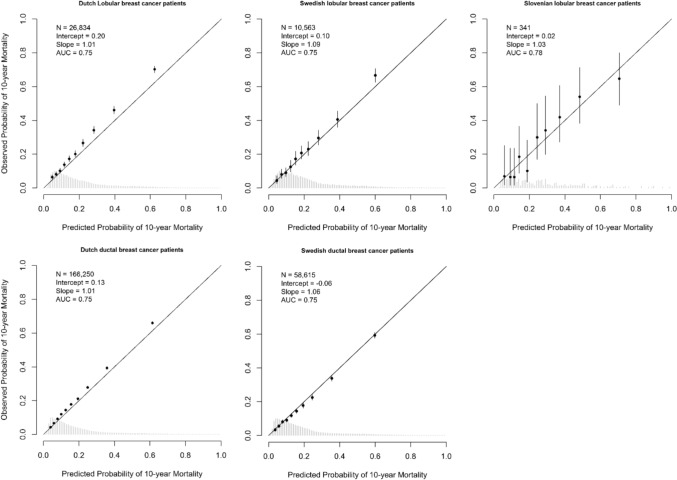
Calibration plots for Dutch (left), Swedish (middle), and Slovenian (right) patients with invasive lobular breast cancer (top row) and invasive ductal breast cancer (bottom row*), showing 10-year overall mortality predictions using PREDICT Breast v3.1. *N*, number of patients included in the external validation set; AUC, area under the curve at 10 years. The intercept and slope (ideally 0 and 1, respectively) are measures used to evaluate calibration. The *Y*-axis represents the observed 10-year mortality, and the *X*-axis represents the predicted 10-year mortality. Each dot indicates the average for a decile of predicted probabilities, with accompanying 95% confidence intervals. *For the Slovenian cohort, we only had access to patients with invasive lobular breast cancer, not those with ductal breast cancer

For patients with ILC, discrimination was comparable to the national populations (Netherlands AUC = 0.75 with 95% CI 0.74–0.76; Sweden AUC = 0.75 with 95% CI 0.73–0.77), and the Slovenian data set (AUC = 0.78 with 95% CI 0.71–0.83). Calibration plots demonstrated underestimation in the Dutch (intercept 0.20; slope 1.01), Swedish (intercept 0.10; slope 1.09), and Slovenian ILC patients (intercept 0.02; slope 1.03; Fig. 2). Calibration plots for 5 years showed similar results (Fig. S4).

**Fig. 3:**
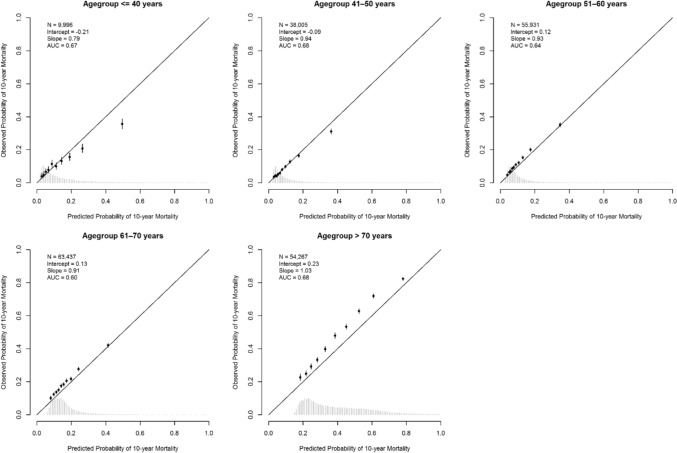
Calibration plots stratified by age group for 10-year overall mortality in Dutch breast cancer patients using PREDICT Breast v3.1. *N*, number of patients included in the external validation set; AUC, area under the curve at 10 years. The intercept and slope (ideally 0 and 1, respectively) are measures used to evaluate calibration. The *Y*-axis represents the observed 10-year mortality, and the *X*-axis represents the predicted 10-year mortality. Each dot indicates the average for a decile of predicted probabilities, with accompanying 95% confidence intervals

**Fig. 4:**
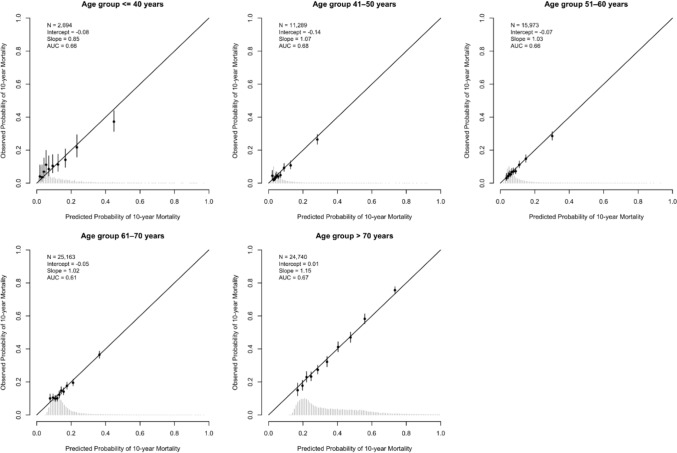
Calibration plots stratified by age group for 10-year overall mortality in Swedish breast cancer patients using PREDICT Breast v3.1. *N*, number of patients included in the external validation set; AUC, area under the curve at 10 years. The intercept and slope (ideally 0 and 1, respectively) are measures used to evaluate calibration. The *Y*-axis represents the observed 10-year mortality, and the *X*-axis represents the predicted 10-year mortality. Each dot indicates the average for a decile of predicted probabilities, with accompanying 95% confidence intervals

For patients aged 40 years or younger, PREDICT overestimated mortality risk, particularly in high-risk groups (Netherlands: intercept − 0.21, slope 0.79; Sweden: intercept − 0.08, slope 0.85; Figs. 3, 4), with comparable findings for 5-year overall mortality, but less pronounced (Fig. S5A, B). Stratified by ER status, miscalibration amongst Dutch patients aged 40 years or younger was more pronounced in ER-negative compared to ER-positive tumours (ER^−^: intercept − 0,51, slope 0.84; ER^+^: intercept 0.05, slope 0.85; Fig. S6A), with comparable findings observed in the Swedish cohort (ER^−^: intercept − 0.51, slope 0.84; ER^+^: intercept 0.05, slope 0.85; Fig. S6B). Additionally, for patients with young triple-negative tumours, miscalibration was observed in both cohorts (Netherlands: intercept − 0.50, slope 1.05; Sweden: intercept − 0.19, slope 0.67; Fig. S7A, B).

PREDICT was generally well calibrated for patients between 40 and 70 years of age. Above 70, mortality was somewhat underestimated in the Netherlands (intercept 0.23, slope 1.03; Fig. 3), and more accurate for patients with ER-negative tumours (intercept 0.11, slope 0.97) than for ER-positive cases (intercept 0.25, slope 1.04; Fig. S6A). In Sweden, for all patients aged > 70 years, mortality risks were well calibrated (ER^−^ intercept − 0.03, slope 1.12; ER^+^ intercept − 0.01, slope 1.13; Fig. S6B). When assessing discrimination within separate age subgroups, the AUC dropped to levels between 0.60 and 0.69 (Figs. 3, 4, Table S3), reflecting the importance of age when predicting mortality.

## Discussion

We validated the PREDICT Breast v3.1 tool in national cohorts from the Netherlands and Sweden and in subgroups of patients with invasive lobular carcinoma (ILC) and those aged 40 years or younger. The model showed excellent calibration of mortality risks in the overall Swedish national cohort and slight underestimation in the overall Dutch national cohort. For ILC, mortality was consistently underestimated in the Dutch, Swedish, and Slovenian cohorts, although overall calibration remained acceptable. In patients aged 40 years or younger, the model was somewhat miscalibrated and overestimated mortality risk in high-risk groups. Overall, these findings support its suitability for clinical use, where PREDICT supports shared decision-making by providing estimates of both survival outcomes and expected treatment benefit. However, further improvements are recommended for the ILC and young patient subgroups.

### National populations

PREDICT v3.1 demonstrated good calibration and discrimination in the national cohorts, consistent with recent external validation studies [28]. However, we observed a slight underestimation of mortality in the Netherlands, compared with a near-perfect calibration in Sweden. Although hormone therapy is incorporated in PREDICT, differences in treatment implementation, adherence, or recording between countries may influence the correspondence between predicted and observed outcomes and could partly contribute to the calibration differences. Hormone therapy use was higher in Sweden (73%) than in the Netherlands (51%). In the UK development dataset, use ranged from 40 to 60% depending on the region [7]. As hormone therapy uptake in the Netherlands falls within this range, differences in uptake alone are unlikely to fully explain the observed calibration pattern. Background mortality may also contribute, as life expectancy is higher in Sweden [29], but further analyses focussing on breast cancer-specific survival and background mortality are needed to test this hypothesis. The most notable difference between the national cohorts was the higher proportion of screen-detected cases in Sweden. This likely reflects national screening policies, with screening starting at 40 years of age in Sweden compared to 50 in the Netherlands. Despite this, the age-at-diagnosis distribution did not indicate earlier diagnoses in Sweden. However, since screen detection is already included as a predictor in the model, such differences are unlikely to fully explain the observed calibration discrepancy between the two populations.

### ILC patients

Although questions have been raised about the applicability of the PREDICT tool for patients with ILC and no previous validation studies have specifically focused on this subgroup, we observed reasonable discrimination and calibration, supporting its use for 5- and 10-year predictions. A possible explanation for the better-than-expected calibration is that previously observed differences in chemotherapy benefit between invasive ductal carcinoma (IDC) and ILC are largely attributable to the higher prevalence of ER-positive and HER2-negative tumours in ILC, rather than histological type itself [30]. As ER and HER2 status are included in the model, these underlying factors are already accounted for in the predictions. Nevertheless, calibration could be further improved, as underestimation of mortality is still observed, most pronounced in the Netherlands (intercept 0.20).

### Young breast cancer patients

For patients aged 40 years and younger, PREDICT v3.1 shows miscalibration, particularly by overestimating mortality risk for young patients at high risk. This trend is seen in both countries and is more pronounced in patients with ER-negative and triple-negative tumours. Similar findings have been reported in other external validation studies of earlier PREDICT versions [15, 30]. This miscalibration appears to be age group specific and may be related to the fact that, in younger patients, mortality is more likely to be predominantly breast cancer related, whereas in older patients, background mortality plays a larger role. It may also reflect their limited representation in the development cohorts, as well as biological differences in tumour behaviour and treatment response. Further research, including competing risk modelling, is needed to refine predictions. Yet accurate prediction is particularly important for younger patients, for whom life expectancy and the long-term consequences of treatment are crucial considerations in shared decision-making.

### Evolution of treatment recommendations and future research

When PREDICT was developed, all patients who had received neoadjuvant therapy were excluded [17]. However, the increasing use of neoadjuvant chemotherapy has led to questions regarding adjuvant therapy recommendations in this group. In the current online version of the tool (PREDICT v3.1), patients are advised to input tumour size measured prior to neoadjuvant chemotherapy. This variable, however, was not available in our dataset, making validation in this context impossible. In patients receiving neoadjuvant chemotherapy, using pathological tumour size confirmed the expected underestimation and underscored the need for validation using pre-treatment size (Fig. S8). In addition, more treatment strategies have evolved over time, with changes in chemotherapy regimens and the introduction of novel therapies such as CDK4/6 inhibitors [31, 32]. These advancements are not yet reflected in PREDICT, underscoring the importance of continuously updating the model to remain relevant for modern clinical practice. Future research should focus on improving model accuracy for specific subgroups and maintaining alignment with evolving treatment options and diverse patient populations. Priorities include validating in patients receiving neoadjuvant therapy, improving modelling of age effects, and improving representation of underrepresented groups in development cohorts. Genomic markers like Mammaprint have modestly improved 5-year breast cancer mortality predictions [25], and future studies should evaluate their impact on long-term outcomes. Finally, whilst PREDICT provides survival estimates based on average treatment effects of adjuvant therapies, generally considered constant on the relative risk scale across subgroups [33, 34], future research could explore potential interactions with specific variables[35].

## Strengths and limitations

A major strength of this study is the use of large, population-based registries from the Netherlands and Sweden, covering multiple years of data and including all surgically treated breast cancer patients in the respective countries. Another key strength is the focus on ILC, a subgroup for which the applicability of current prediction tools has not been well established. Moreover, data from three countries could be used, although the Slovenian cohort consists of previously collected data from an earlier treatment period, which may limit representativeness for current clinical practice.

Certain limitations should be acknowledged. In this study, no distinction could be made between breast cancer-specific mortality and overall mortality, unlike in the PREDICT tool, making it difficult to determine whether performances reflect breast cancer-specific or background mortality. Additionally, smoking was not available, and therefore a fixed smoking prevalence of 15% based on Dutch population data was assumed. Having smoking information available would change the absolute individual mortality estimates, but the impact on overall discrimination would only be marginal [36]. Furthermore, data on 15-year survival outcomes are lacking. Such long-term information is especially relevant for patients with ILC, as deviations from average population predictions tend to become more apparent over time [8], and for younger patients, given their longer life expectancy. Finally, input data were not entirely harmonised between the two national cohorts; for example, Ki-67 was only available in the Swedish dataset and its assessment is known to be subject to interobserver variability [37]. Whilst sensitivity analyses suggested that treating Ki-67 as unknown had a minor impact on performance, differences in data availability should be considered when interpreting results across both populations.

## Conclusion

PREDICT Breast v3.1 is generally well calibrated for the evaluated European national populations. Although PREDICT demonstrated reasonable performance for lobular breast cancer patients, calibration could be further improved. For patients aged 40 and younger, an overestimation of mortality risk in high-risk patients was observed. Improved modelling of age effects may enhance the overall predictive performance of the tool for younger patients.

## Supplementary Information

Below is the link to the electronic supplementary material.

Supplementary file1 (DOCX 1525 KB)

## Data Availability

We used data from registries and clinical databases in three different countries. The data are owned by the contributing hospitals or registries and are available upon reasonable request, subject to institutional approvals.
