# Supplementary material for: External validation of PREDICT Breast v3.1 for overall survival in international cohorts, including young and invasive lobular subgroups
Source: Breast Cancer Res Treat. 2026 Apr 17;217(2):14. doi: 10.1007/s10549-026-07958-w (PMC13086695; doi:10.1007/s10549-026-07958-w)
Supplement: Supplementary file 1 — Supplementary file1 (DOCX 1525 KB) [file 10549_2026_7958_MOESM1_ESM.docx]

**Supplementary material

Title:** External validation of PREDICT Breast v3.1 for overall survival in international cohorts, including young and invasive lobular subgroups
**Journal:** Breast cancer research and treatment
**Authors:** Elfi M. Verheul, Frank Doornkamp, Iurii Petrov, Sabine Siesling, Hester F. Lingsma, Linetta B. Koppert, Lara W.A. Vreven, Adri C. Voogd, Maria Margarete Karsten, Lea Doppelbauer, Pimrapat Gebert, Narsis Kiani, Simona Borstnar, Paul D. P. Pharoah, Elham Hedayati, Ewout W. Steyerberg, David van Klaveren, on behalf of the 4D PICTURE Consortium#
**Correspondence to**: Elfi M. Verheul, [e.verheul@erasmusmc.nl](mailto:e.verheul@erasmusmc.nl), affiliations: Center for Medical Decision Making, Department of Public Health, Erasmus University Medical Center, Rotterdam & The Netherlands and Dutch Institute for Clinical Auditing, Leiden, The Netherlands.

**Content Supplementary material**
Table S1: Definitions and assumptions of Predict v3.1 predictors and treatments Page 2

Figure S1A, B, C: Distribution of patients per inclusion year per cohort Page 3

Figure S2A, B: Calibration plots 10-year OM stratified for estrogen receptor (ER) status Page 4

Figure S3: calibration plots 5-year overall mortality (OM) national populations Page 5

Figure S4: calibration plots 5-year OM lobular breast cancer patients Page 5

Figure S5A, B: Calibration plots 5-year OM stratified for age groups Page 6

Figure 6A, B: Calibration plots 10-year OM stratified for ER status and agegroup Page 7, 8

Figure 7A, B: Calibration plots 10-year OM stratified for triple negative and agegroup Page 9

Figure S8: Calibration plot 10-year OM: treated with neoadjuvant chemotherapy Page 10

Table S2: TRIPOD checklist Page 11

Table S3: AUC by age group, with 95% confidence intervals Page 12

| **Predictor / Treatment** | **Definition** | **Notes and assumptions** |
| --- | --- | --- |
| **Patient characteristics** | | |
| Age at diagnosis | Continuous: in years | Based on age at primary diagnosis date. |
| Smoker | Categorical: Yes / Never or ex (when quit ≥1 year before diagnosis) | Missing for all patients; assigned mean population value (0.15) based on recent Dutch breast cancer data. |
| Post-menopausal | Categorical: Yes / No / Unknown. Defined as ≥12 months amenorrhea or surgical menopause before diagnosis. | Available in the online tool but not included in risk estimation, it only enables the treatment option Bisphosphonates in the tool. As we do not have information on both of these variables, this was left out the validation study. |
| **Tumour characteristics** | | |
| ER status | Categorical: Positive (≥10%) / Negative (<10%) | Missing values imputed by multiple imputation. |
| PR status | Categorical: Positive / Negative / Unkown | Missing values set to 'Unknown'. |
| HER2/ERRB2 status | Categorical: Positive / Negative/ Unkown | Only HER2 variable available; missing values set to 'Unknown'. |
| Ki-67 status | Categorical: Positive (≥10%) / Negative | Missing values set to 'Unknown'. |
| Invasive tumor size (mm) | Continuous, pathological tumor size in millimeters | Missing values imputed by multiple imputation. |
| Tumor grade | Grade 1 = well differentiated; Grade 2 = moderately differentiated; Grade 3 = poorly/undifferentiated | Missing values imputed by multiple imputation. |
| Detected by screening | Categorical: Yes if detected through national screening program; No if clinically detected; unknown. | Screen detection was registered in the Dutch data as participation in the national screening program; if marked as "no", we assumed symptomatic detection; Missing values, set to 'Unknown'. |
| Positive nodes | Continuous; count of positive lymph nodes | Missing values imputed by multiple imputation. |
| Micrometastases only | Categorical: Yes, if nodal metastases ≤2 mm; No when macrometastases; unknown | Missing values set to 'Unknown'. |
| **Treatment characteristics** | | |
| Radiotherapy | Categorical: Yes / No Includes radiotherapy to the breast | Radiotherapy assumption Netherlands and Sweden: If radiotherapy was given, it was assumed to correspond to 2 Gy for the left breast and 0 Gy for the right breast. For Slovenia, information on the side of the breast was missing; however, assuming an equal probability of having cancer on left or right side, all patients who received radiotherapy were assigned a value of 1 Gy. |
| Adjuvant hormonal therapy | Categorical: Yes / No | Assumed duration of 5 years as per guidelines due to missing data on actual treatment length. |
| Adjuvant targeted therapy (trastuzumab) | Categorical: Yes / No | Only applicable for HER2-positive patients. As trastuzumab data were unavailable in the Slovenian dataset, we assumed that all HER2-positive patients received this treatment. |
| Adjuvant chemotherapy | Categorical: No chemotherapy at all / Standard-dose, anthracycline-based (selects chemotherapy drug regimes such as FEC; fluorouracil, epirubicin and cyclophosphamide) / High dose, anthracyline- or taxane-based (selects chemotherapy drug regimes that contain taxanes) | Chemotherapy assumption: If chemotherapy status was unknown, patients were assumed to have received high-dose, anthracycline- or taxane-based treatment.  First-generation regimens (e.g., CMF) excluded as not supported by PREDICT-v3.1 |
| Bisphosphonates | Binary: Yes / No | Set to zero for all patients due to lack of information in databases. Impact of this assumption was shown not significantly impacting the results before. The impact of this assumption has previously been shown not to significantly affect the results (24). |

Table S1: Predictors and treatment variables of the predict v3.1 tool, including their definitions and assumptions

Supplementary figure 1A: Distribution of inclusion year of breast cancer patients in the Netherlands.

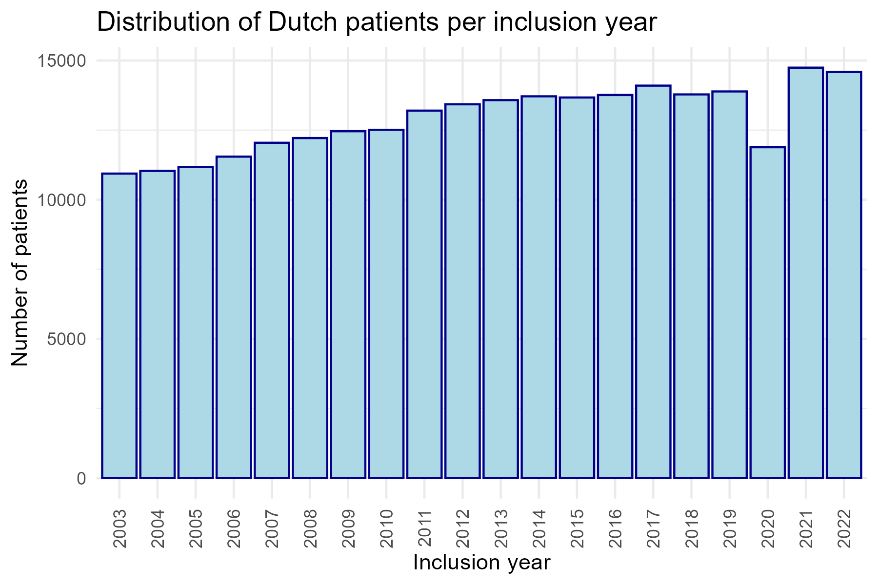


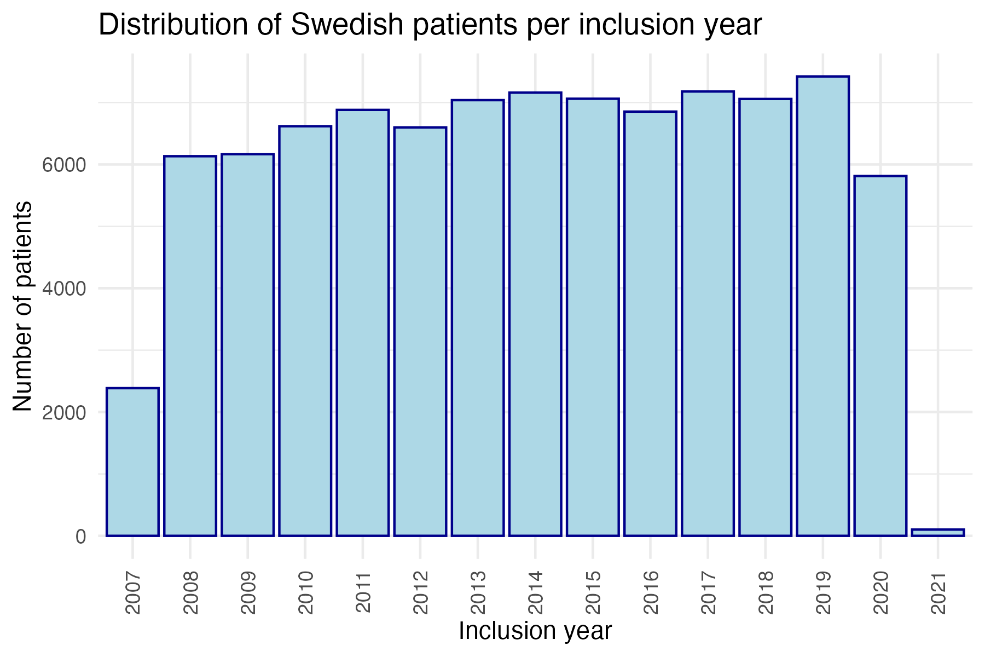


Supplementary Figure 1B: Distribution of inclusion year of breast cancer patients in Sweden.

**
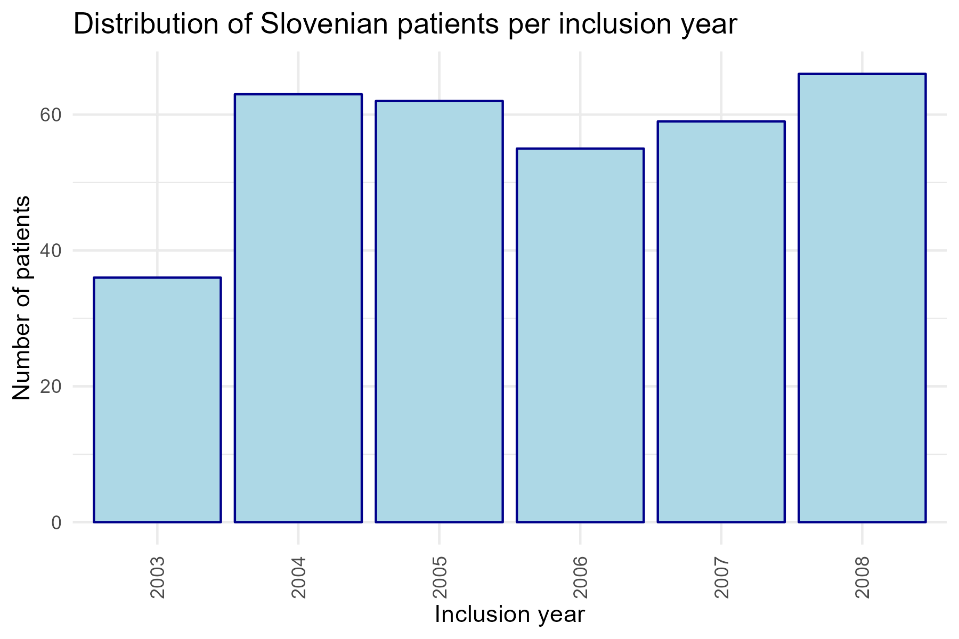
**Supplementary Figure 1C: Distribution of inclusion year of lobular breast cancer patients in Slovenia.


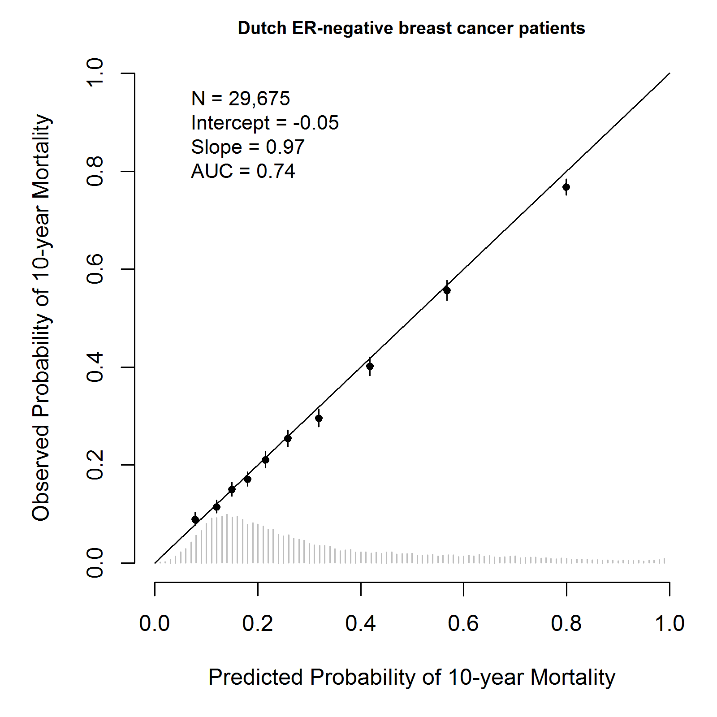

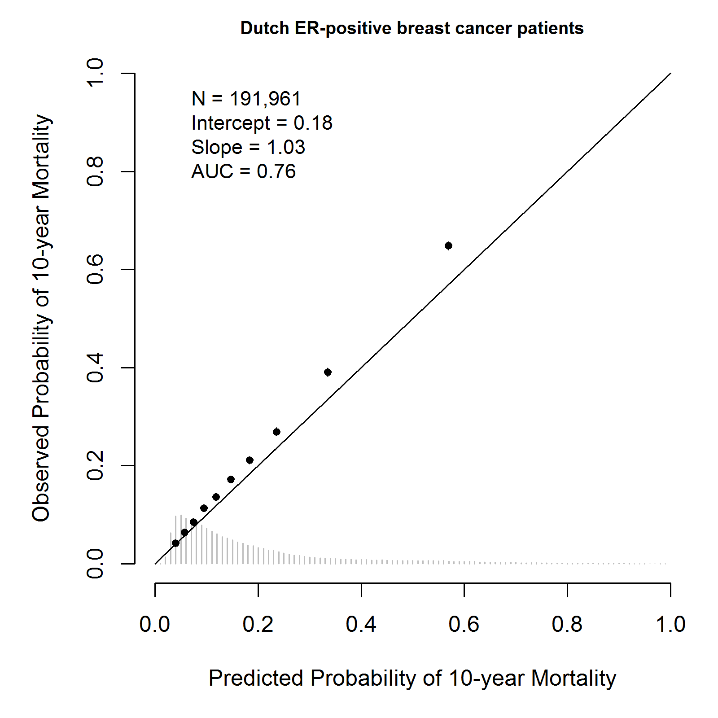
Supplementary Figure 2A: Calibration plots stratified by estrogen receptor (ER) status, for 10-year overall mortality in Dutch breast cancer patients using PREDICT Breast 3.1.


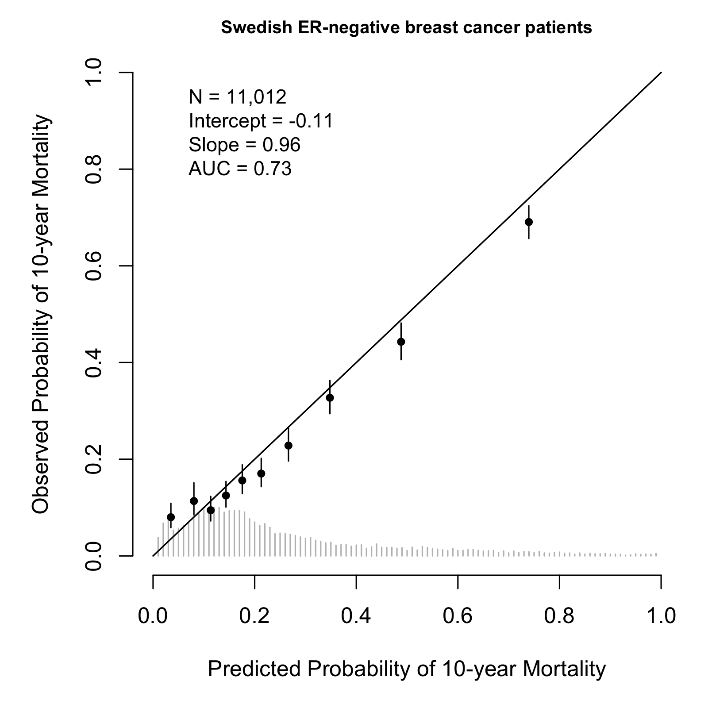

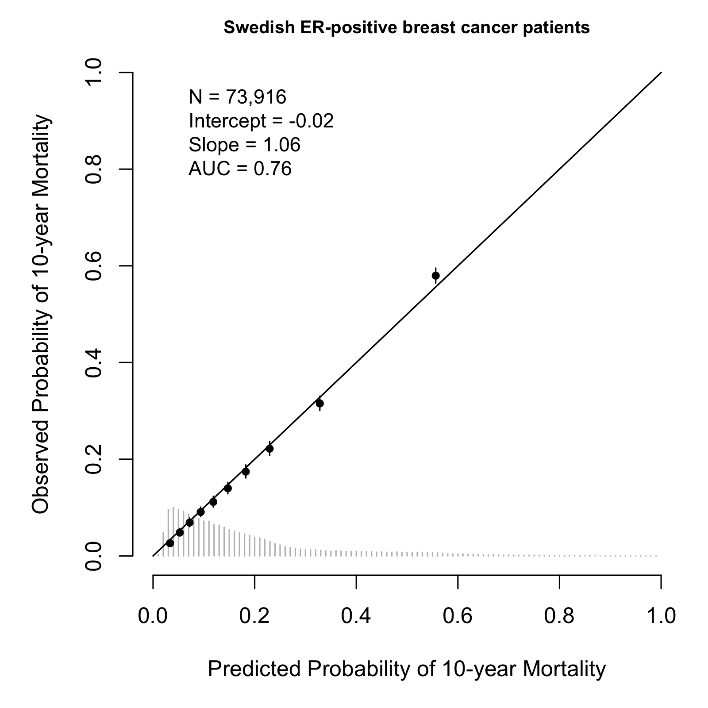
Supplementary Figure 2B: Calibration plots stratified by estrogen receptor (ER) status, for 10-year overall mortality in Swedish breast cancer patients using PREDICT Breast 3.1.

Supplementary figure 3: Calibration plots for the Dutch (left) and Swedish (right) populations,
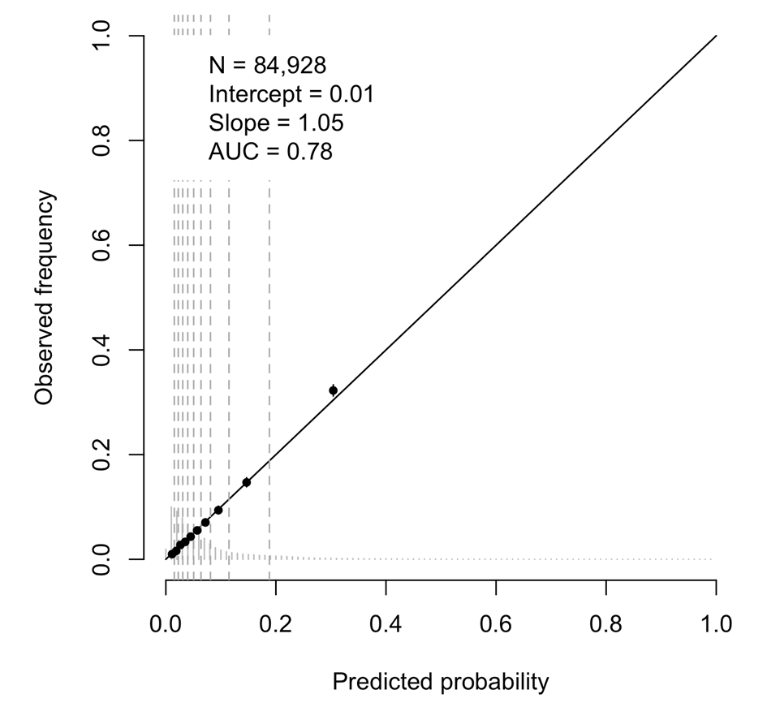

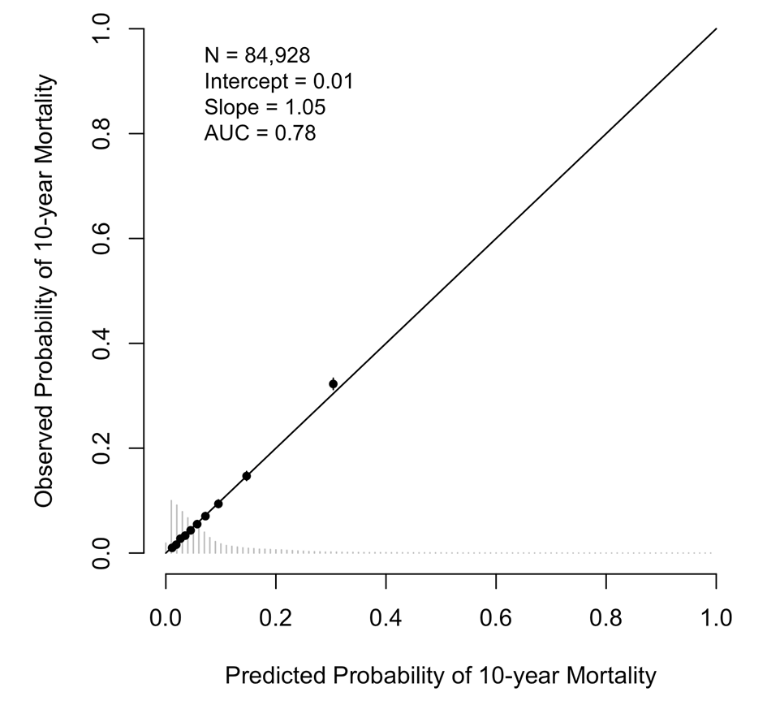

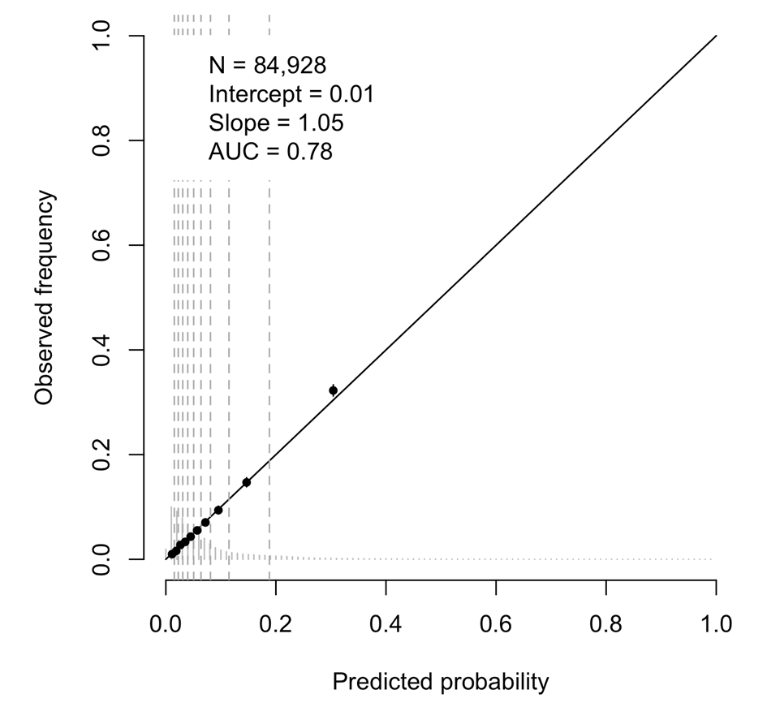

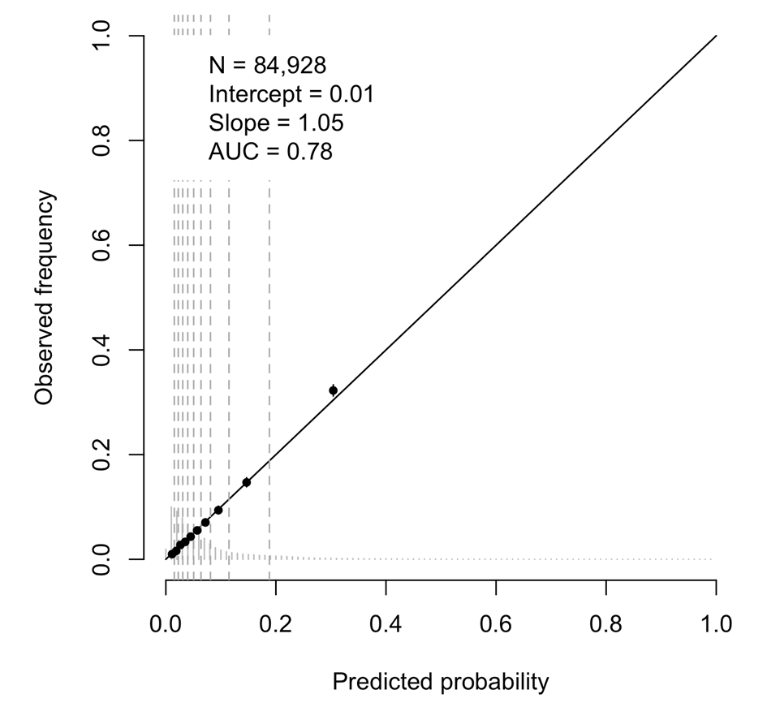

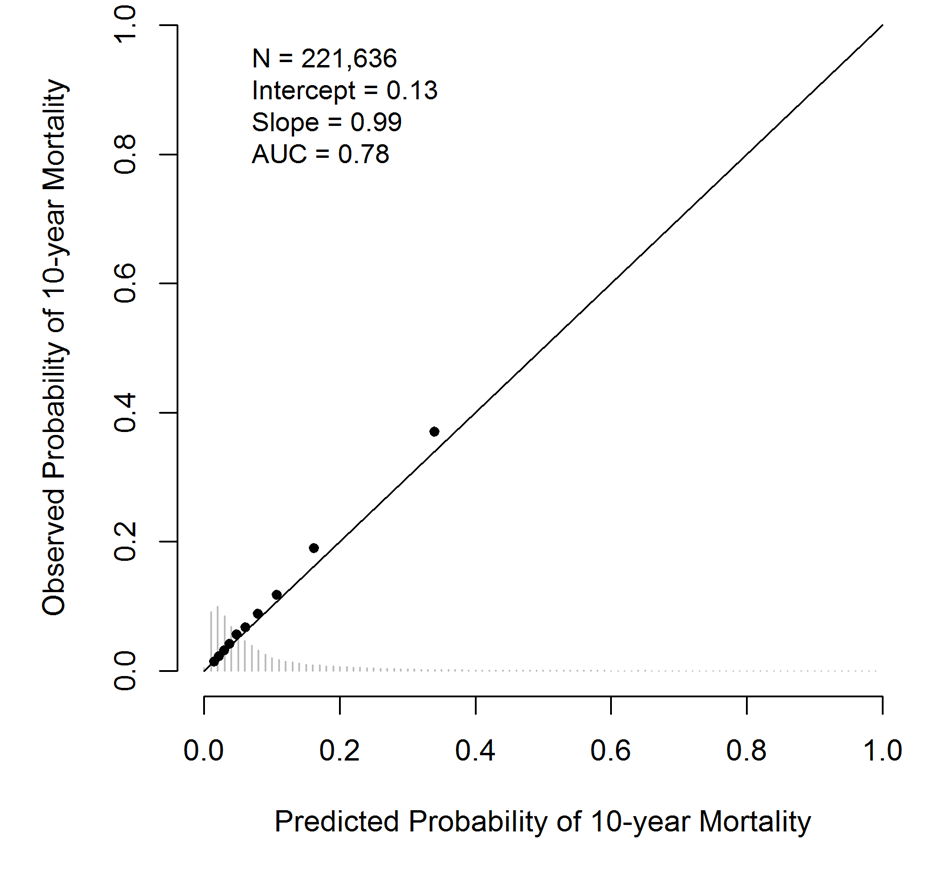

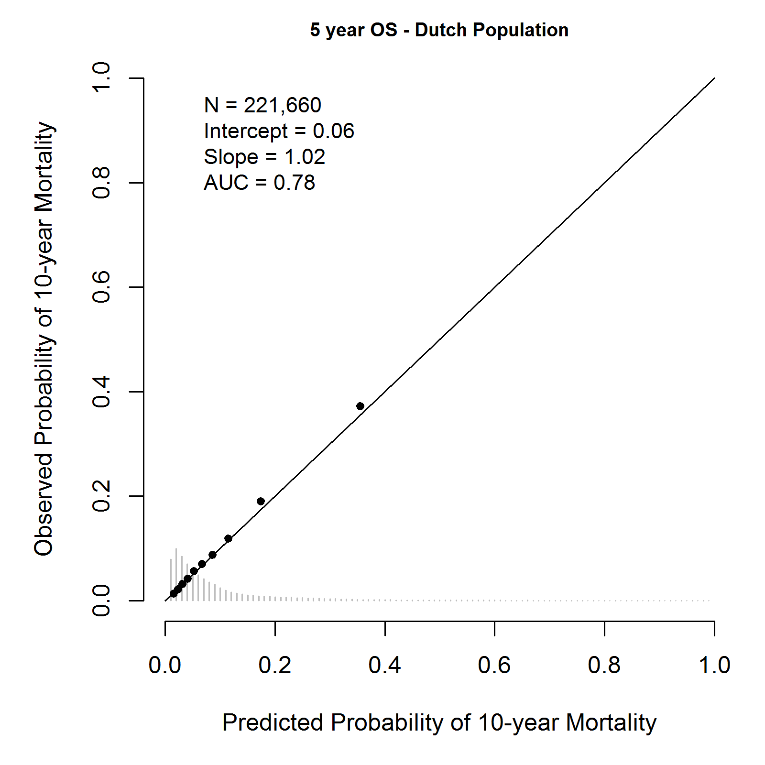

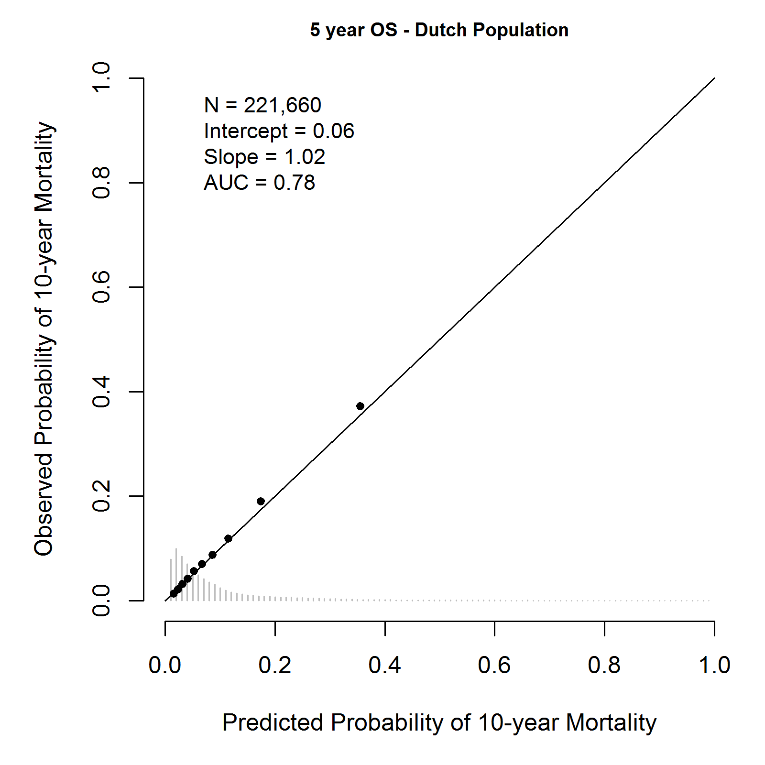
showing 5-year overall mortality predictions using PREDICT Breast 3.1.


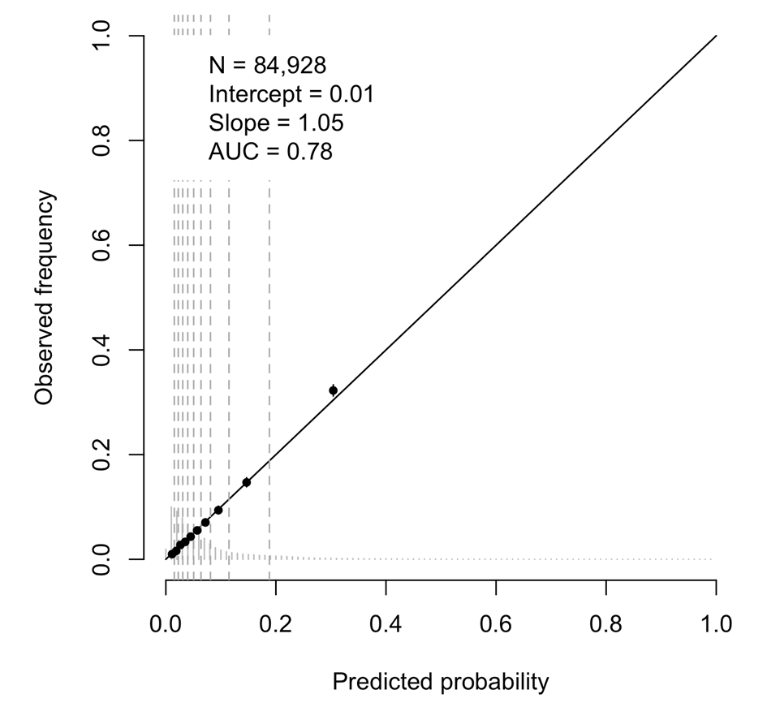

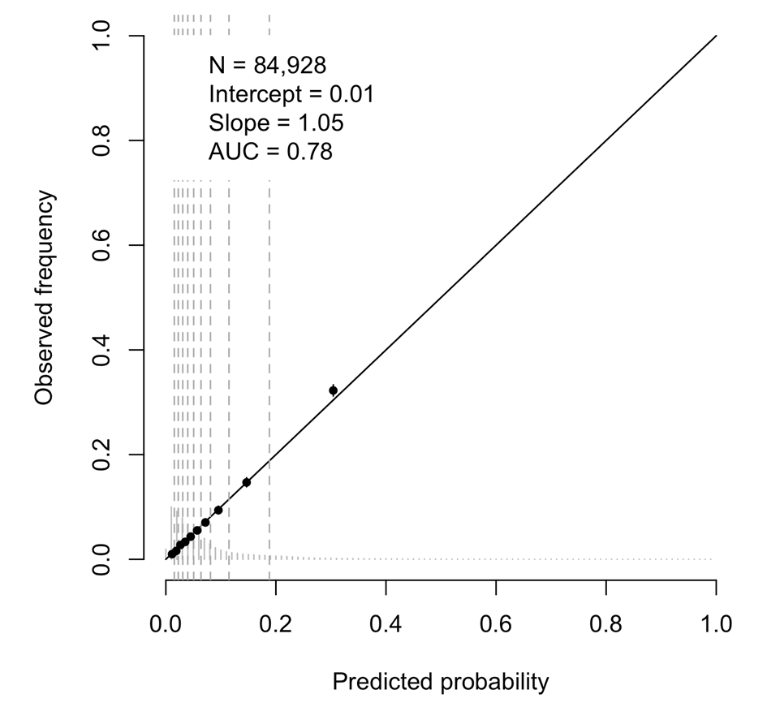

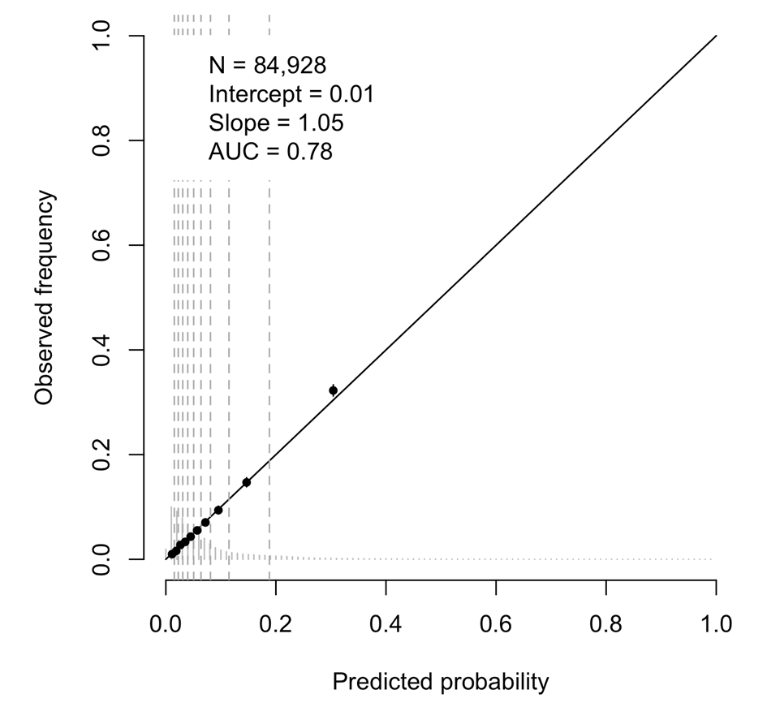

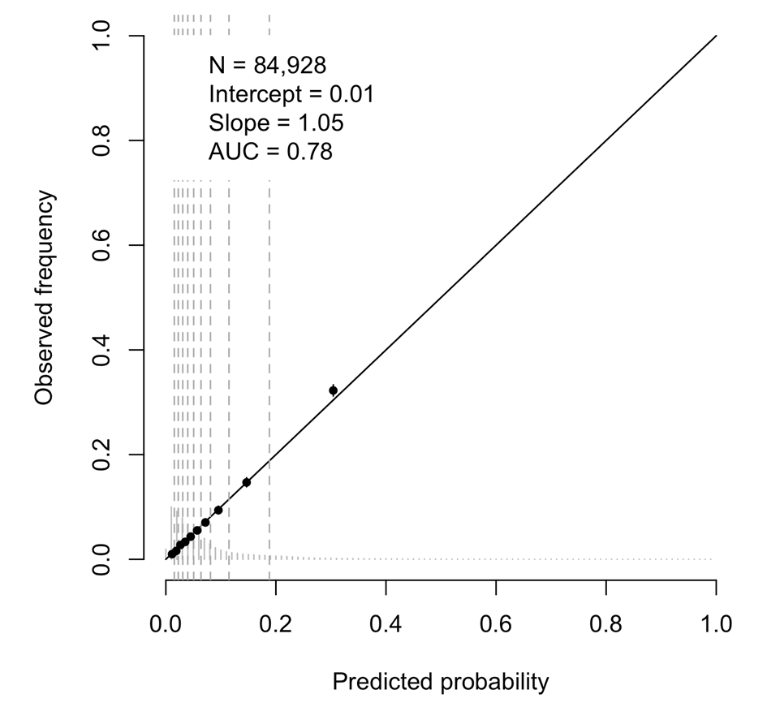
**
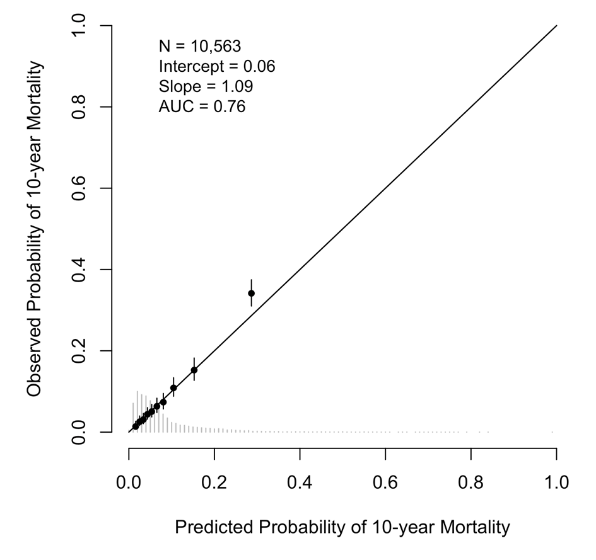
**
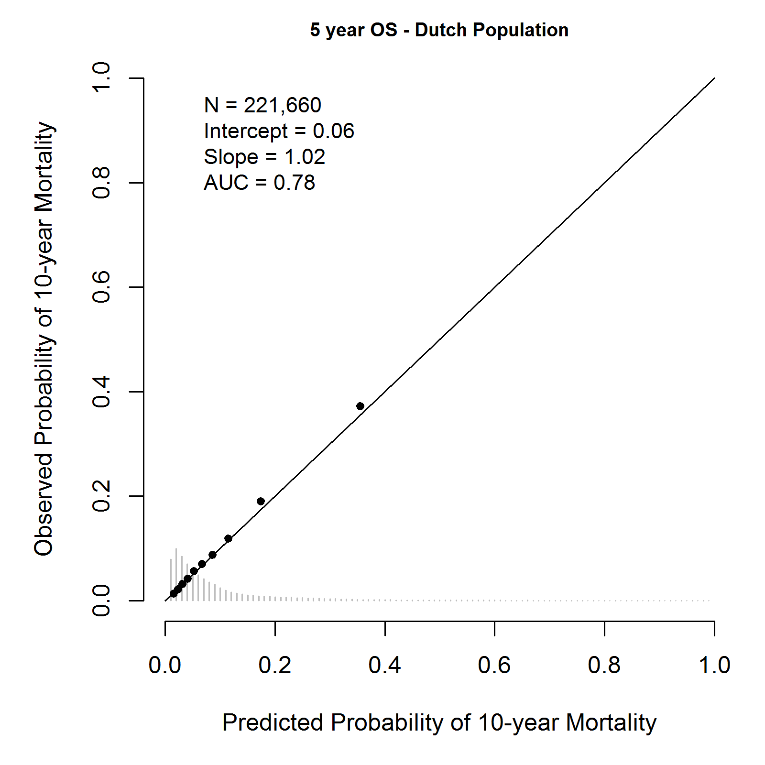

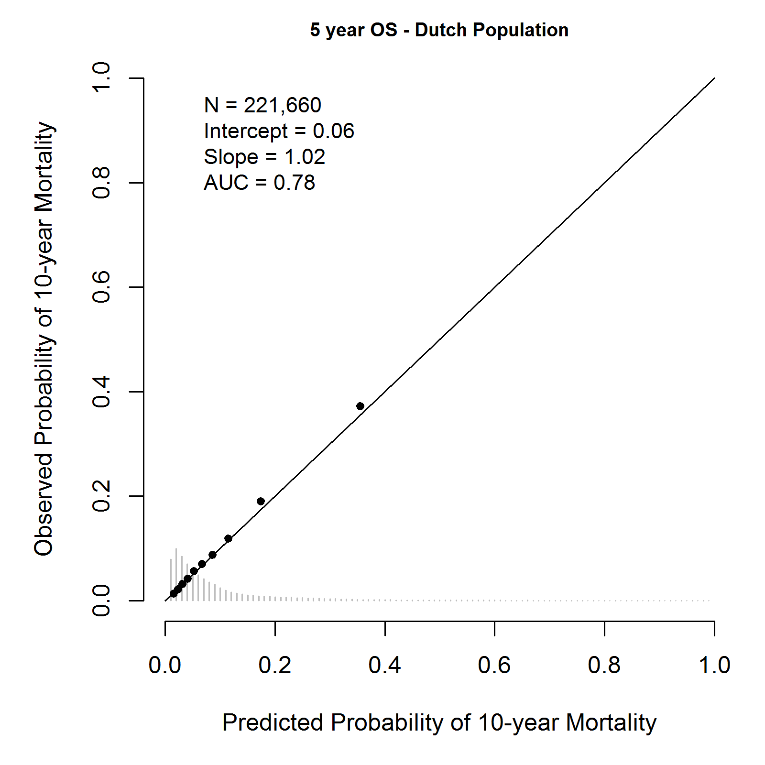

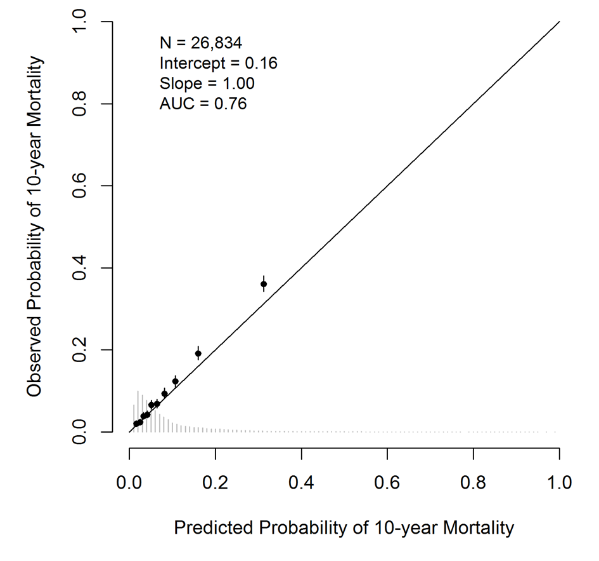

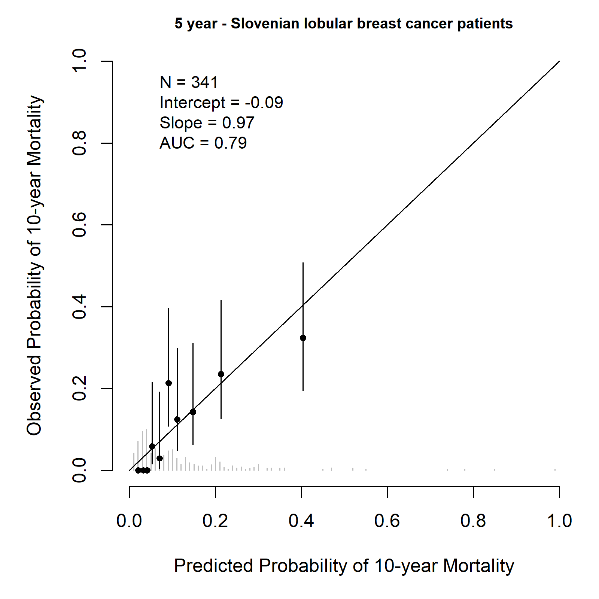

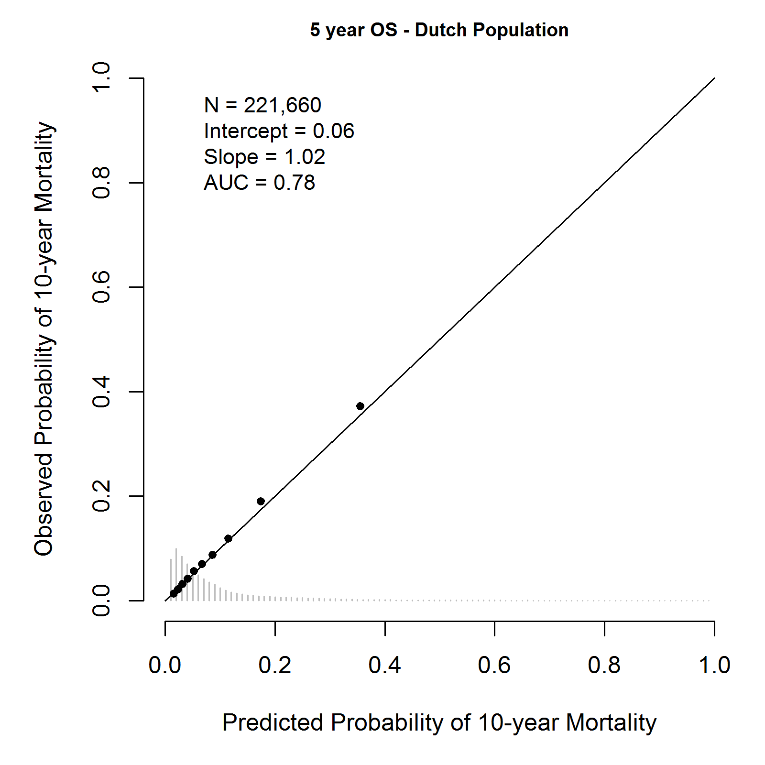

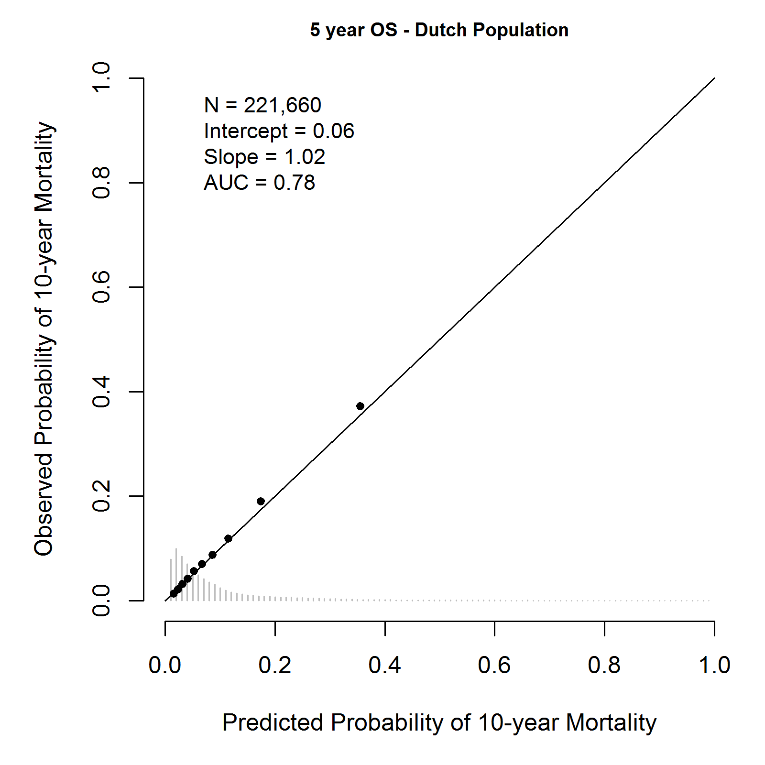
Supplementary figure 4: Calibration plots for Dutch (left), Swedish (middle) and Slovenian (right) lobular breast cancer patients, showing 5-year overall mortality predictions using PREDICT Breast 3.1.

**
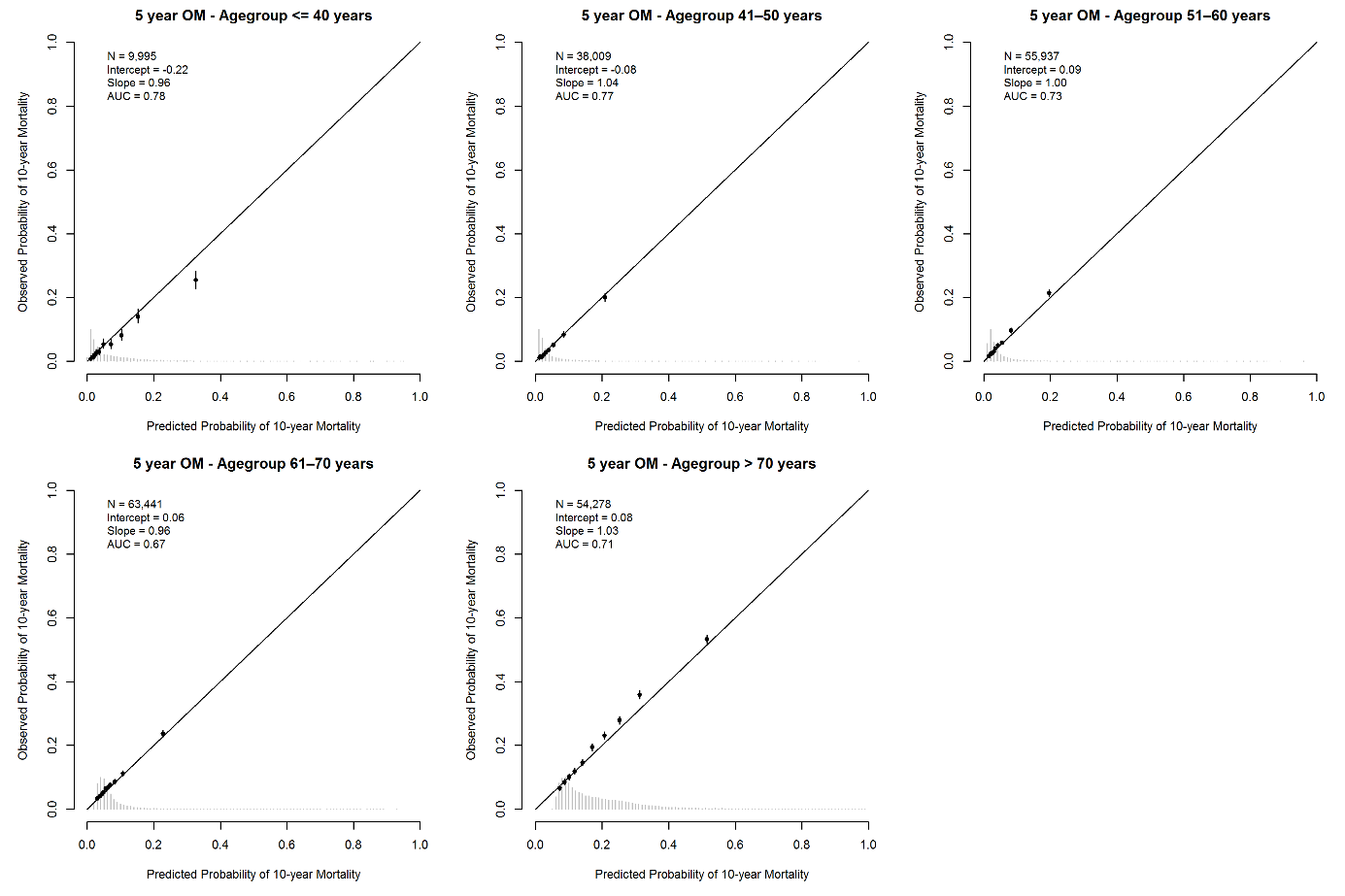

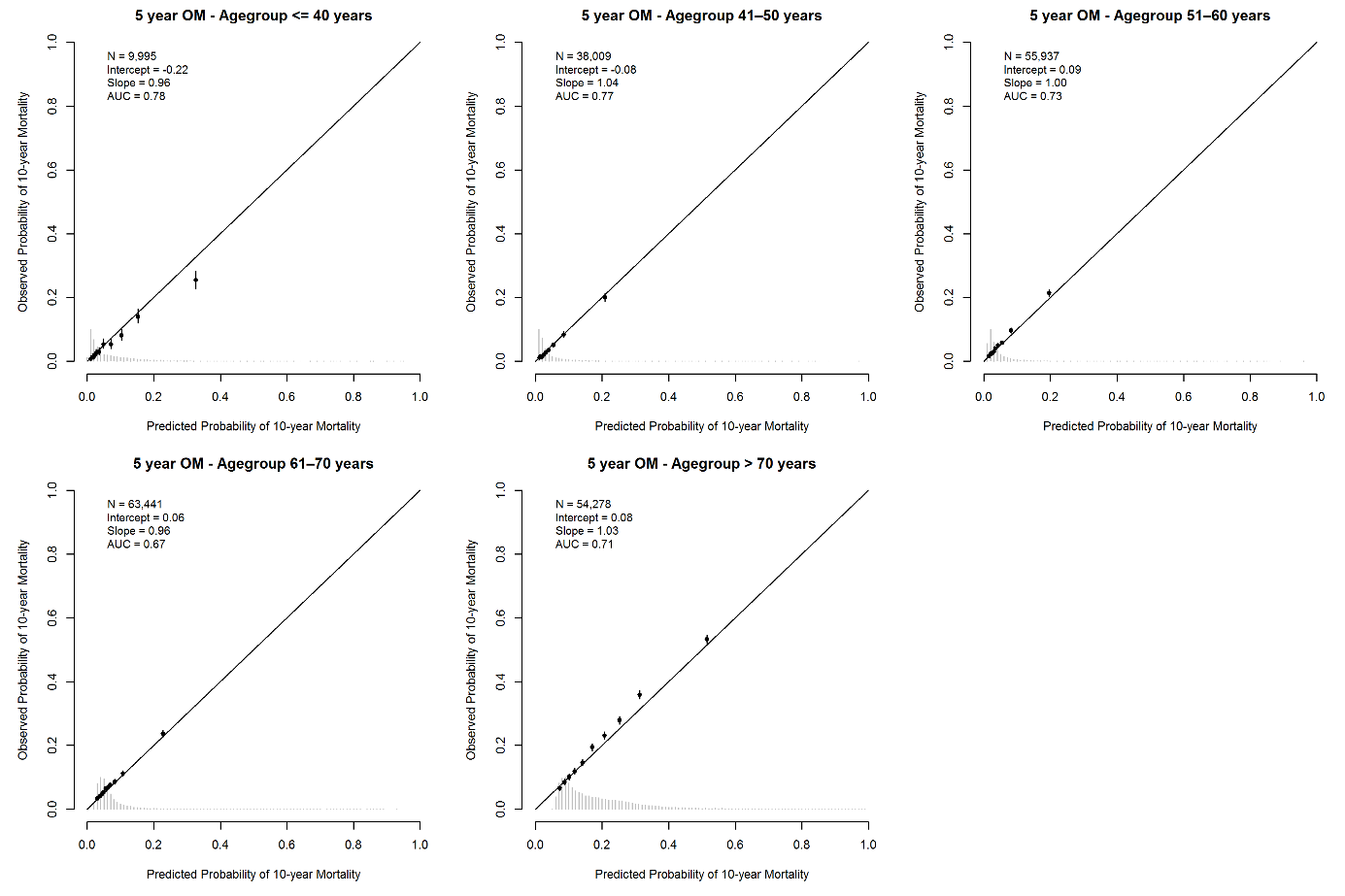

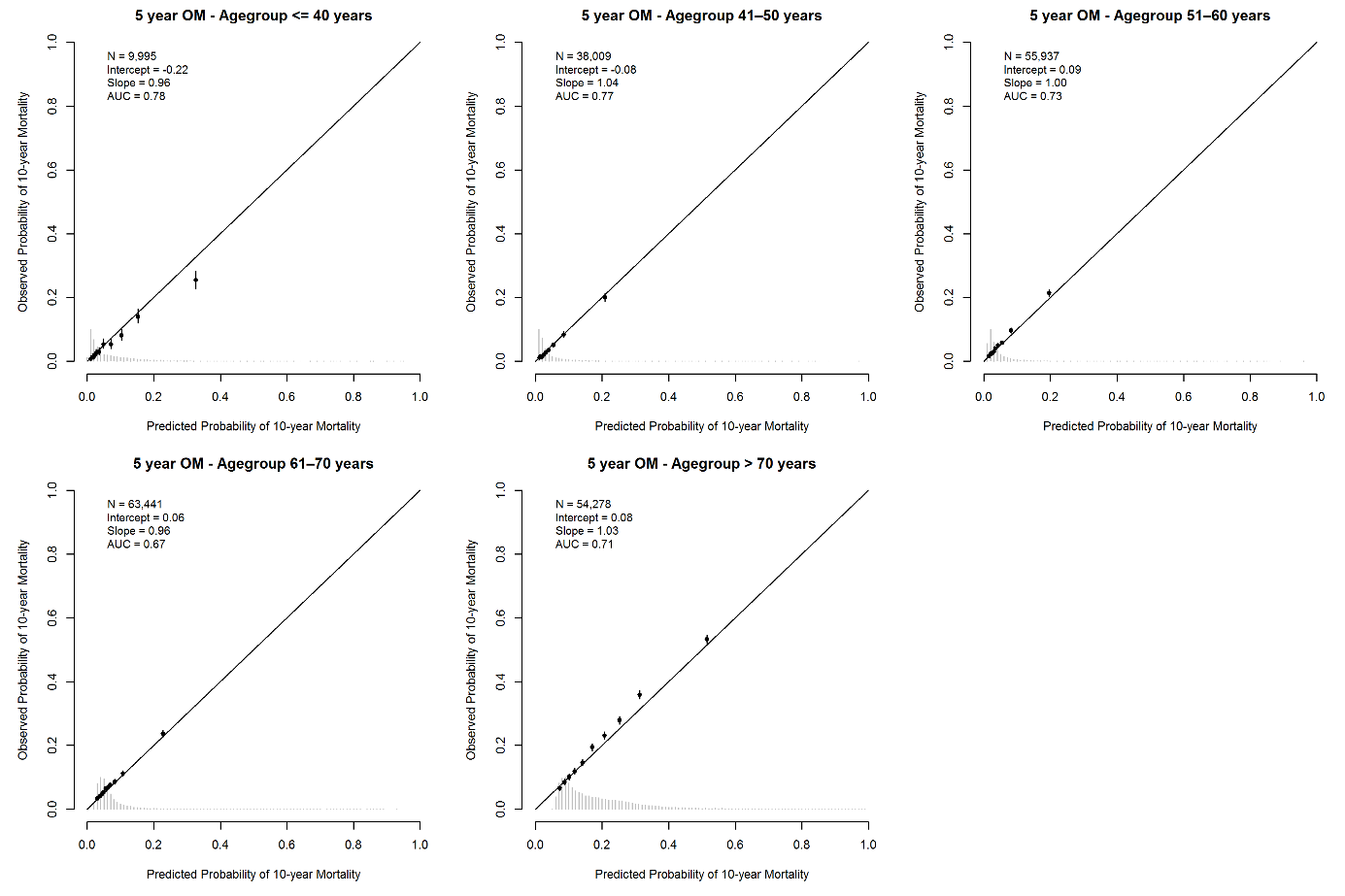

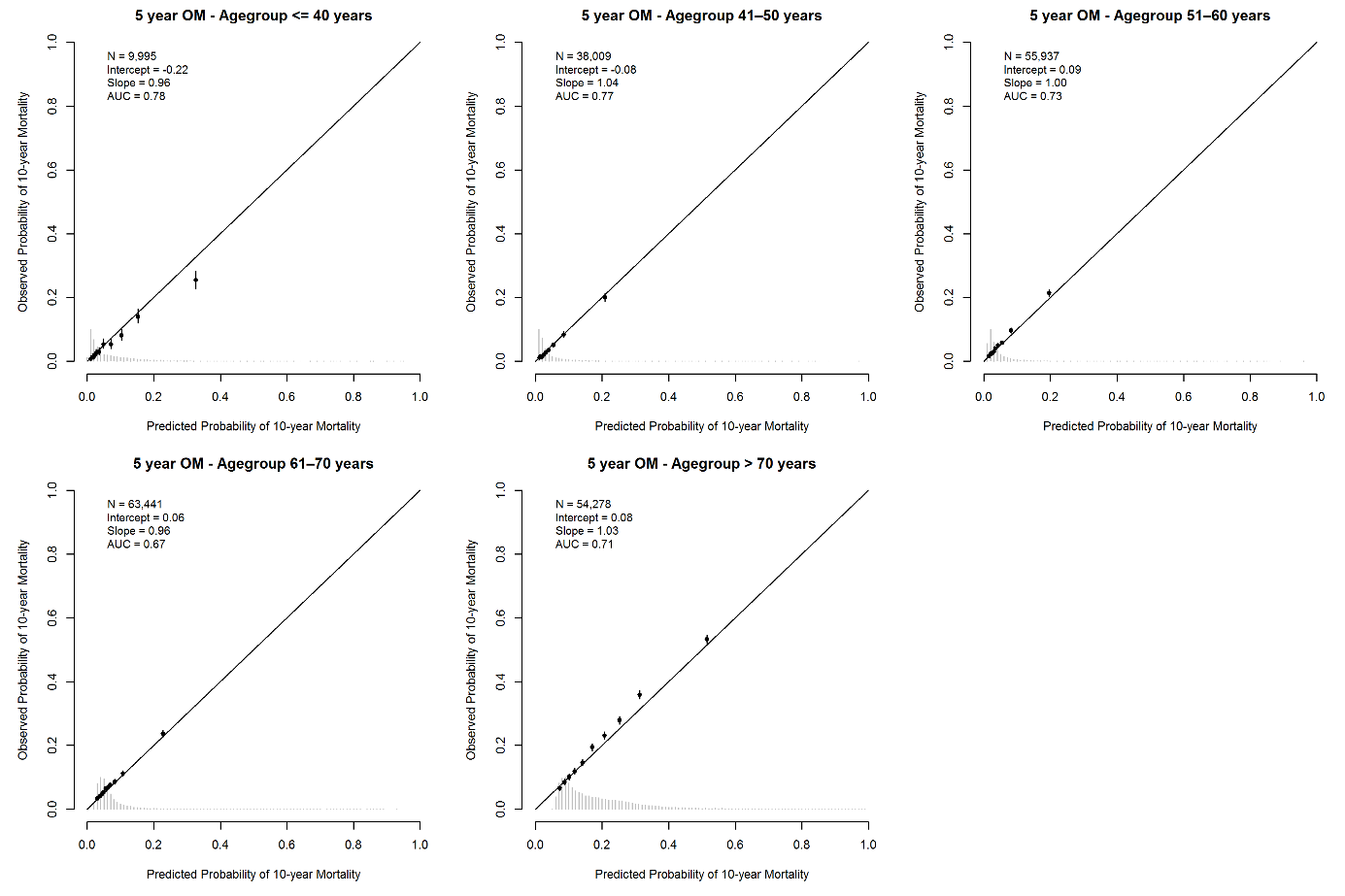

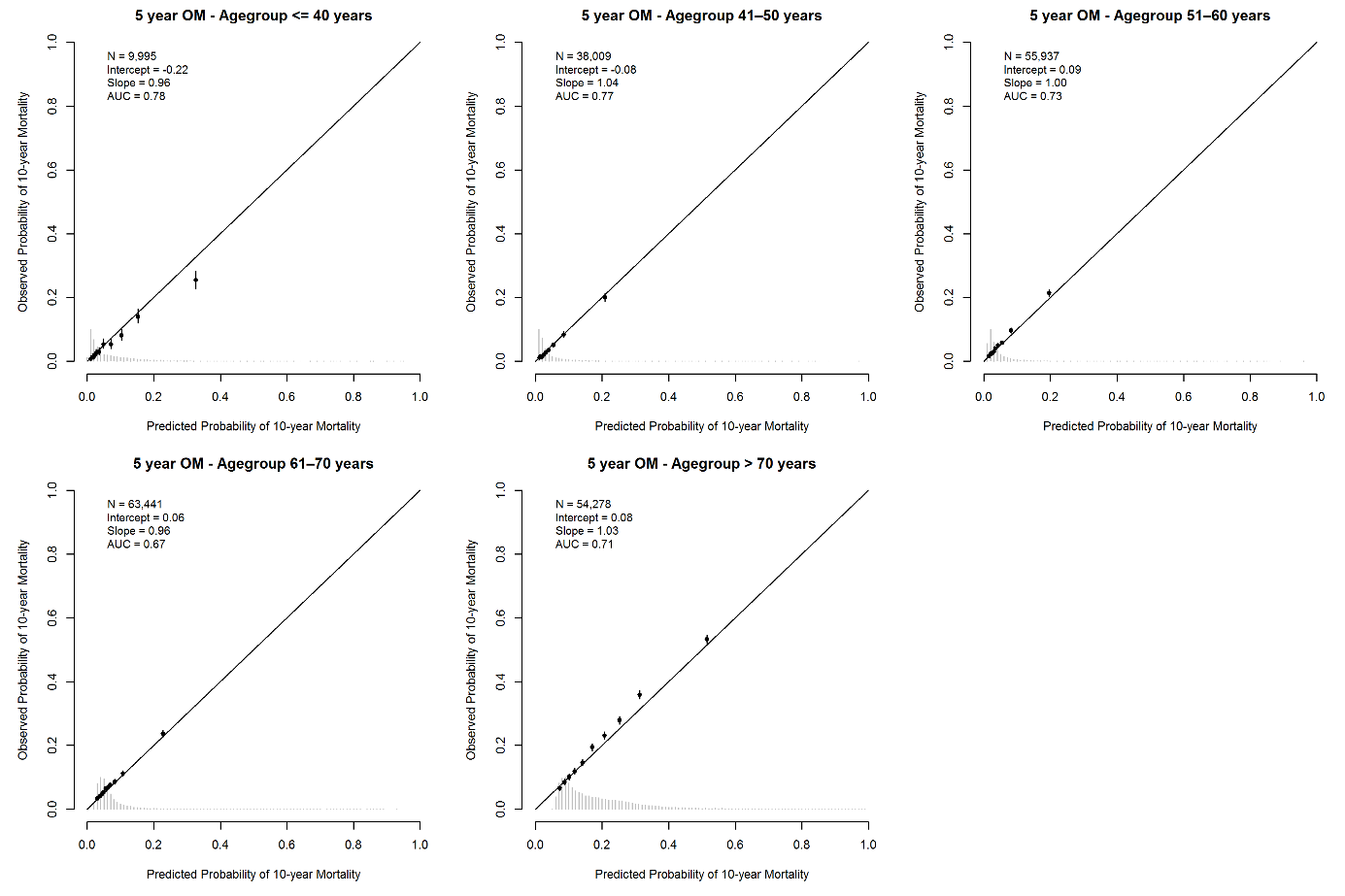
**
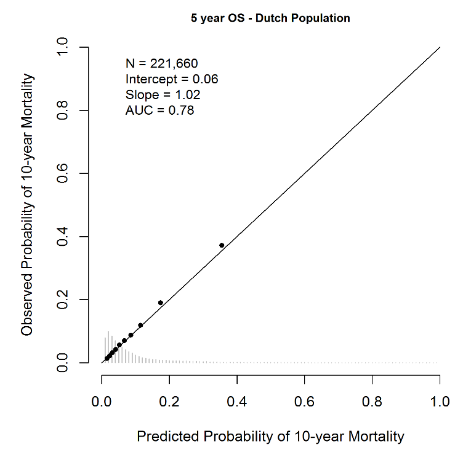

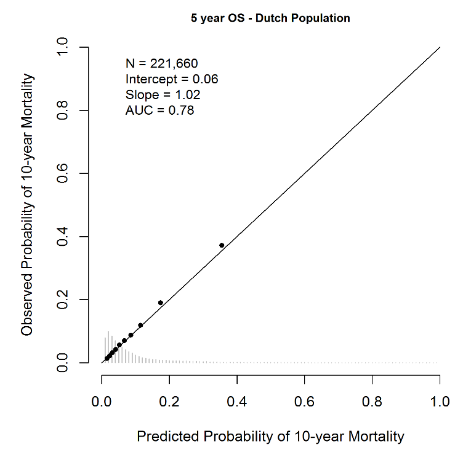

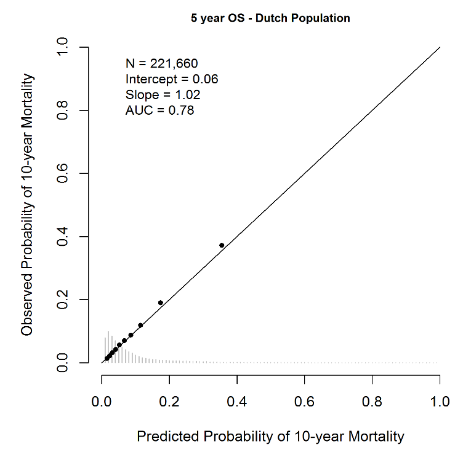

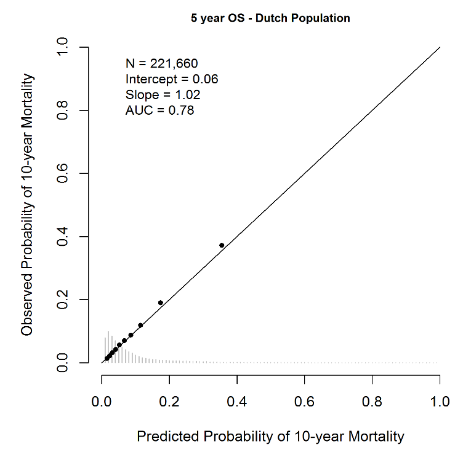

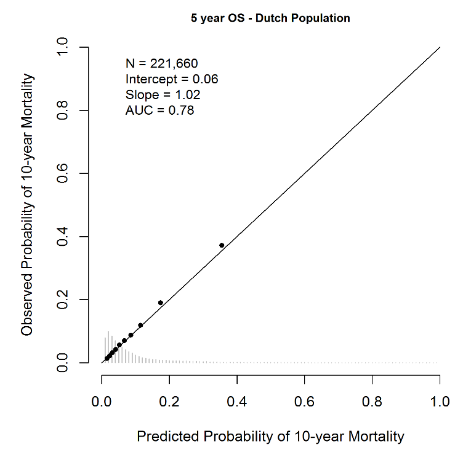

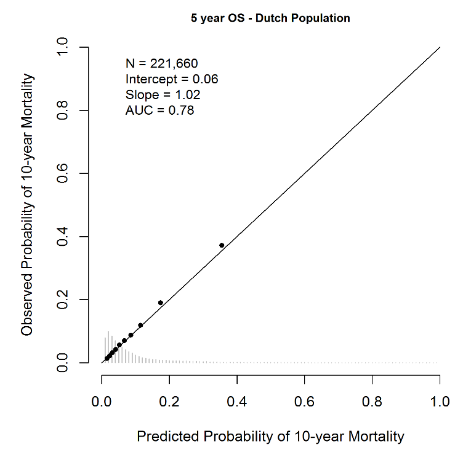

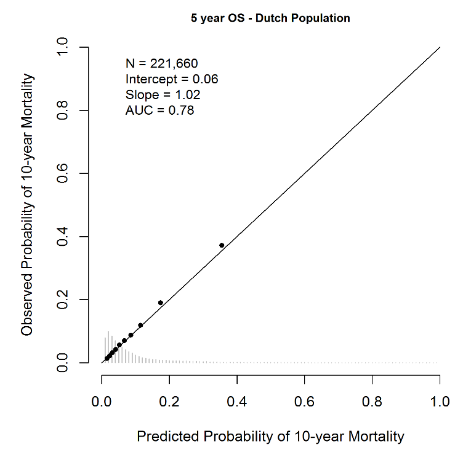
**
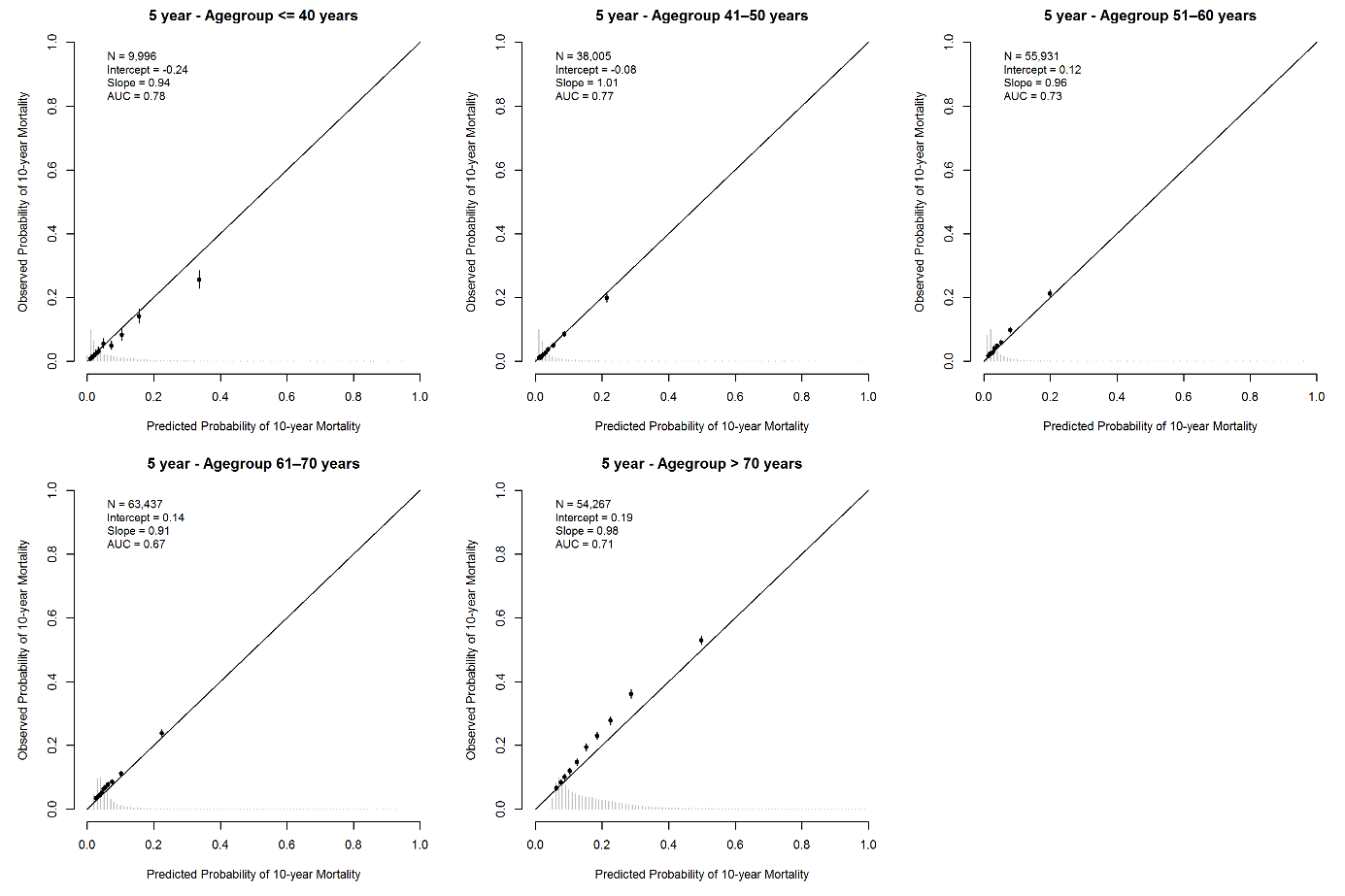
**Supplementary figure 5A: Calibration plots stratified by age group for 5-year overall mortality (OM) in Dutch breast cancer patients using PREDICT Breast 3.1. **
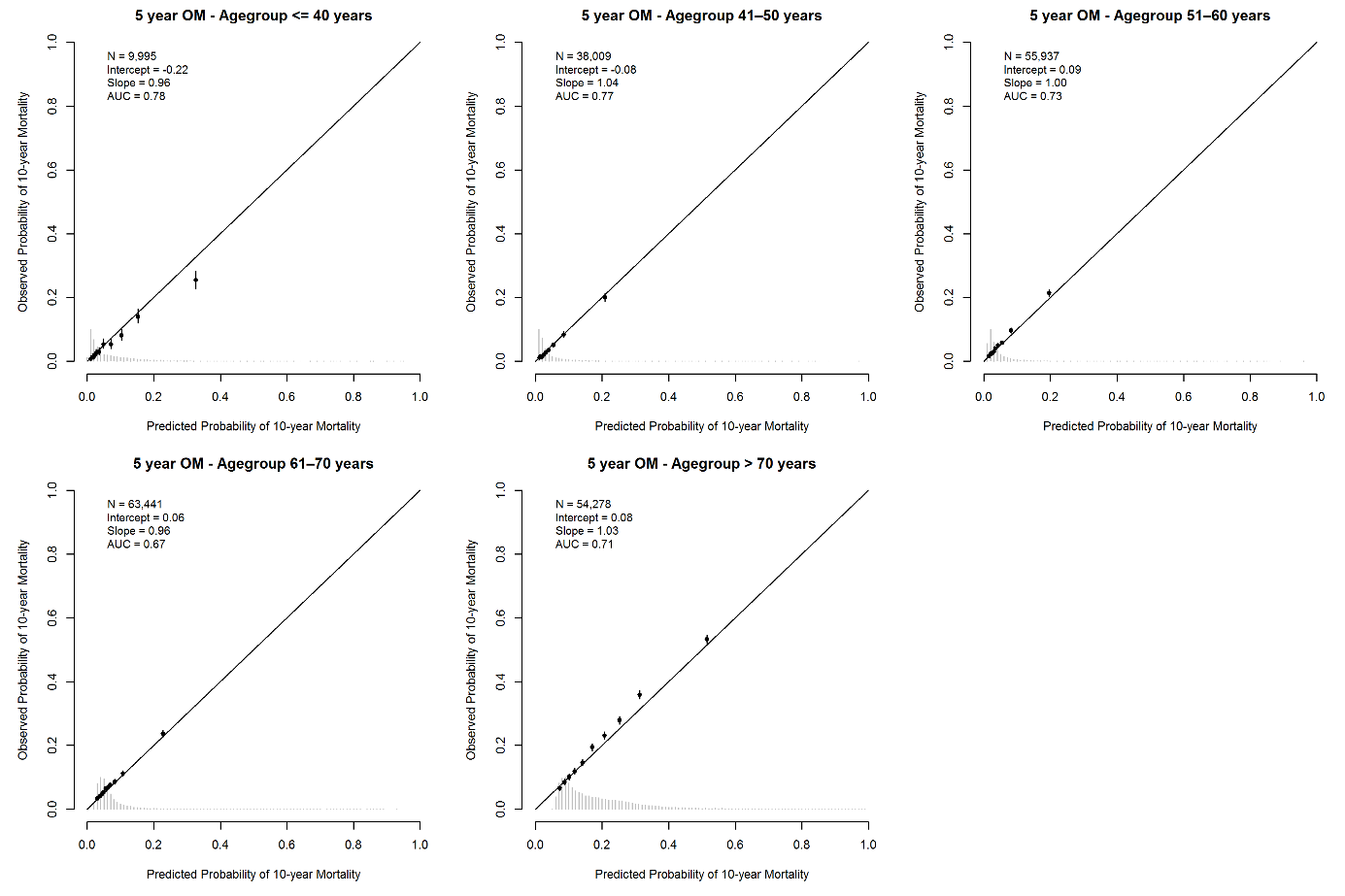

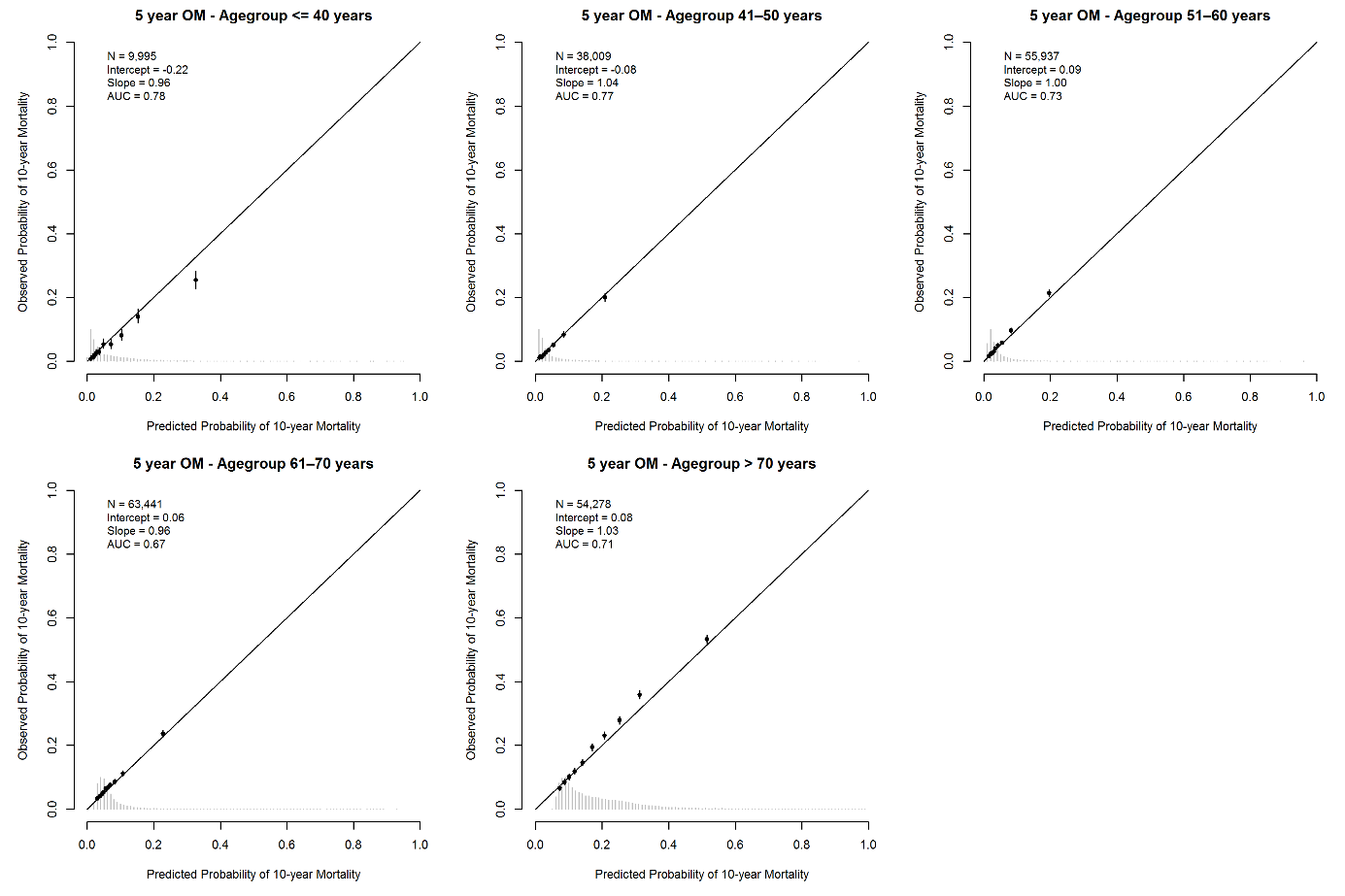
**

Supplementary figure 5B: Calibration plots stratified by age group for 5-year overall mortality (OM) in Swedish breast cancer patients using PREDICT Breast 3.1.

**
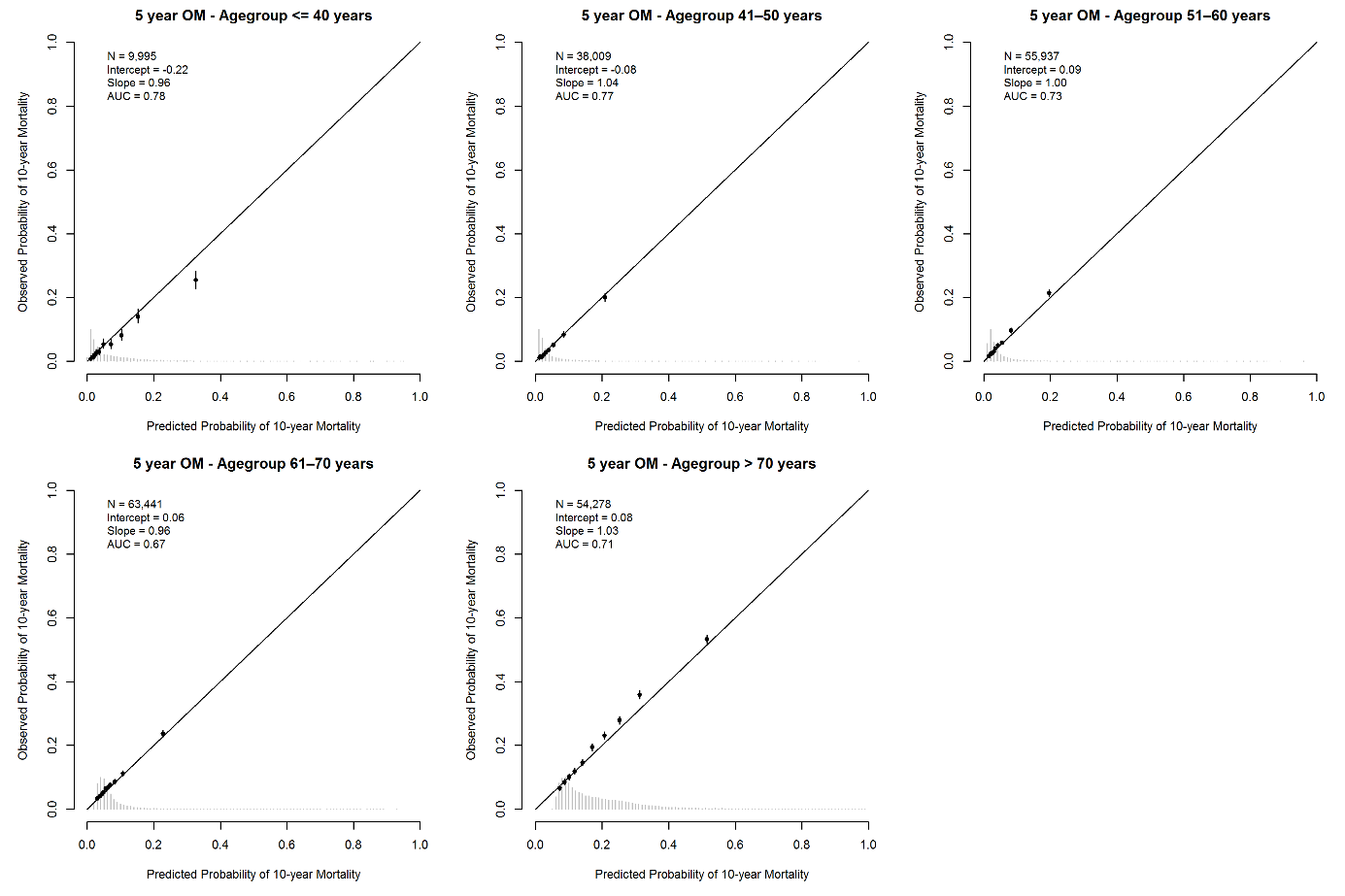

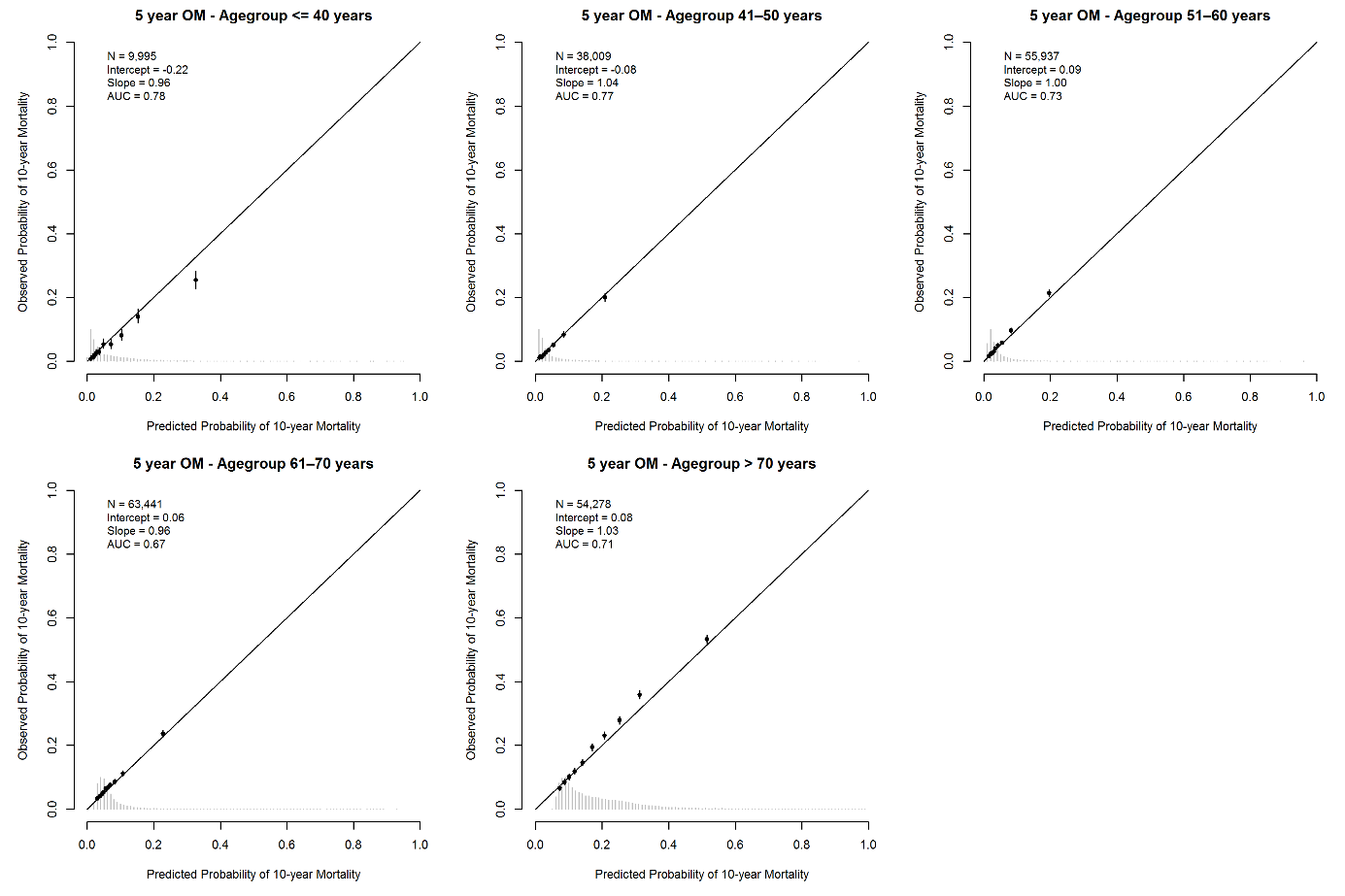
**
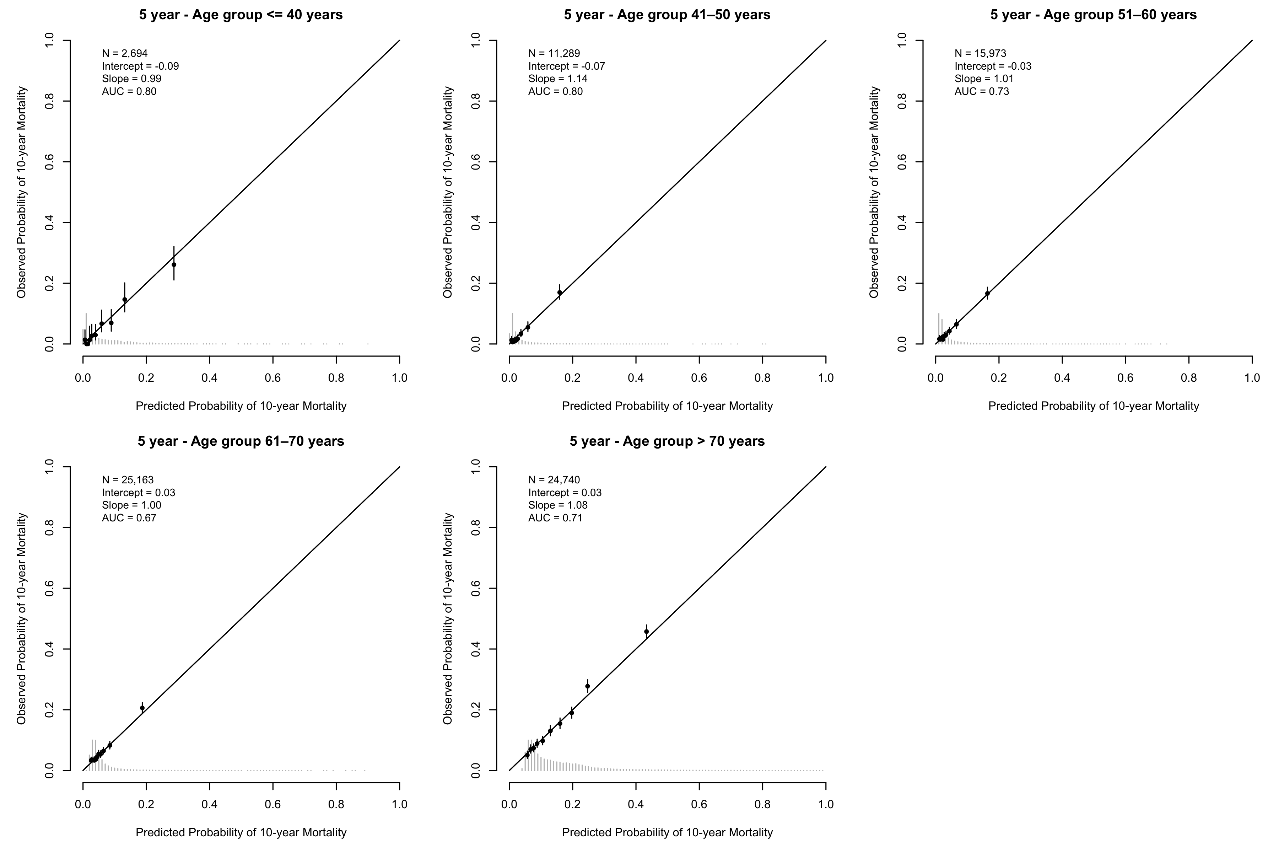


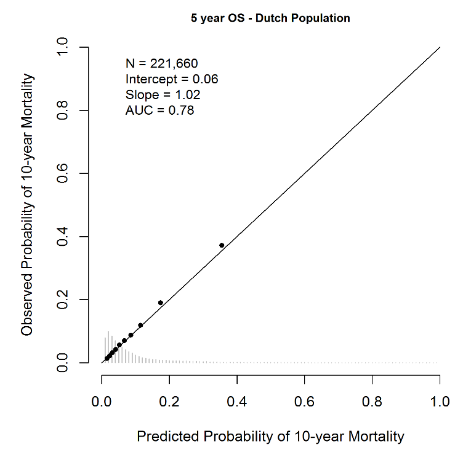

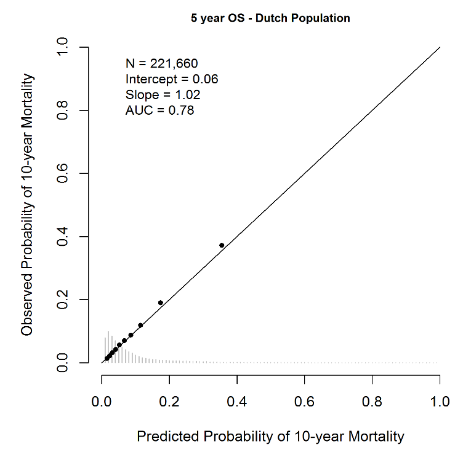

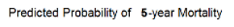

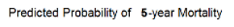

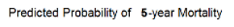

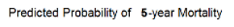

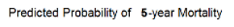


S

Supplementary Figure 6A: Calibration plots stratified by age group and estrogen receptor (ER) status, for 10-year overall mortality in Dutch breast cancer patients using PREDICT Breast 3.1.


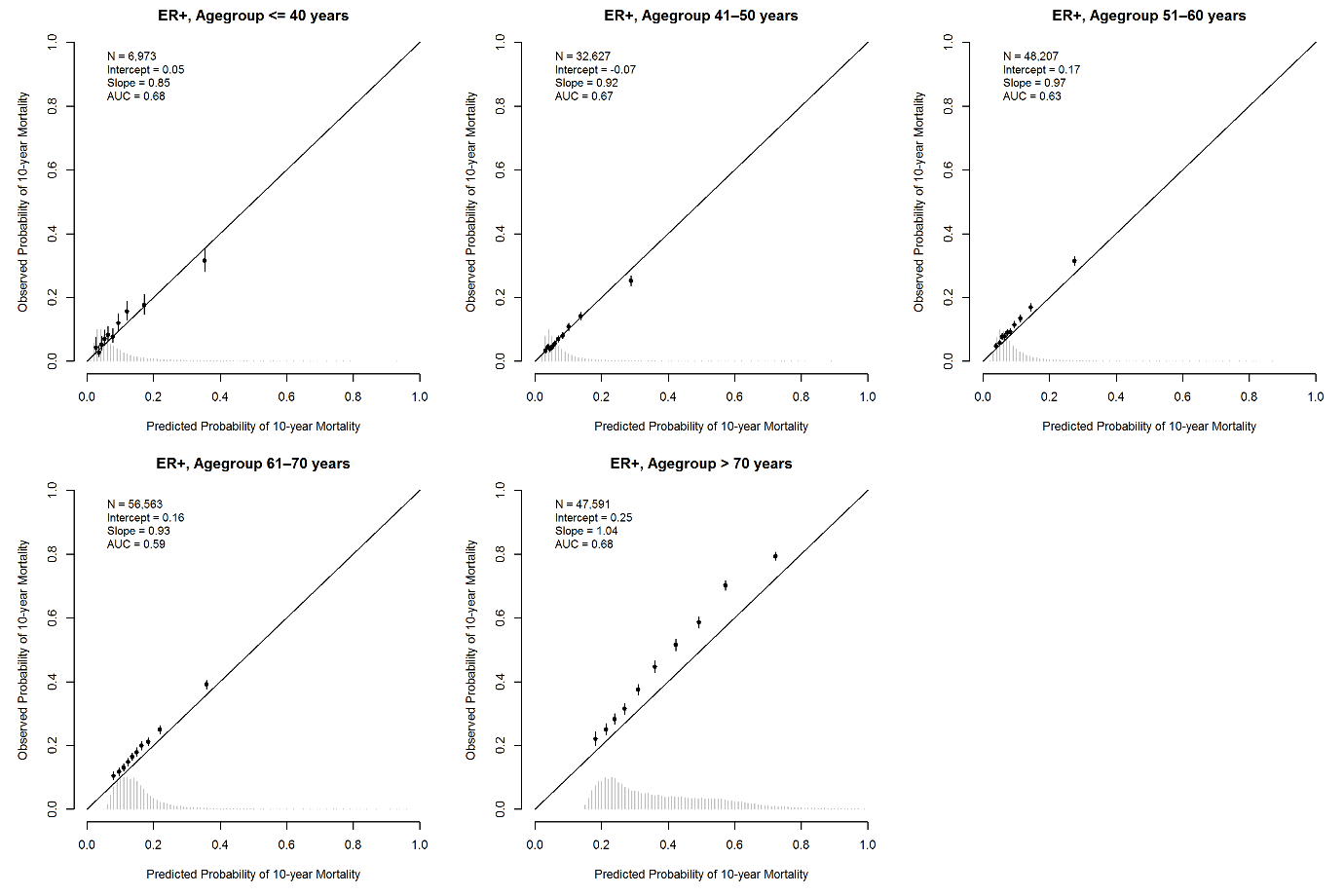


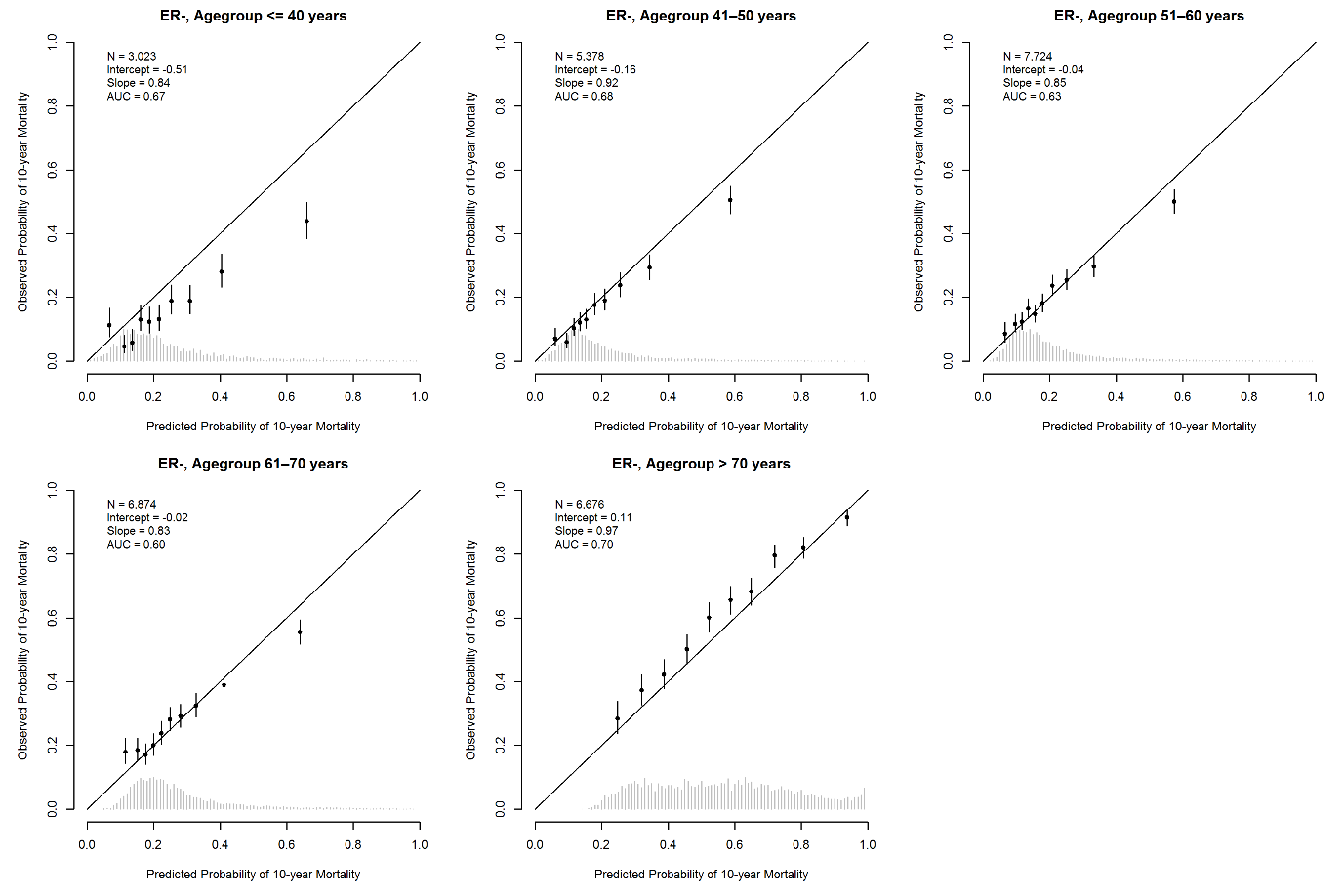


Supplementary Figure 6B: Calibration plots stratified by age group and estrogen receptor (ER) status, for 10-year overall mortality in Swedish breast cancer patients using PREDICT Breast 3.1.


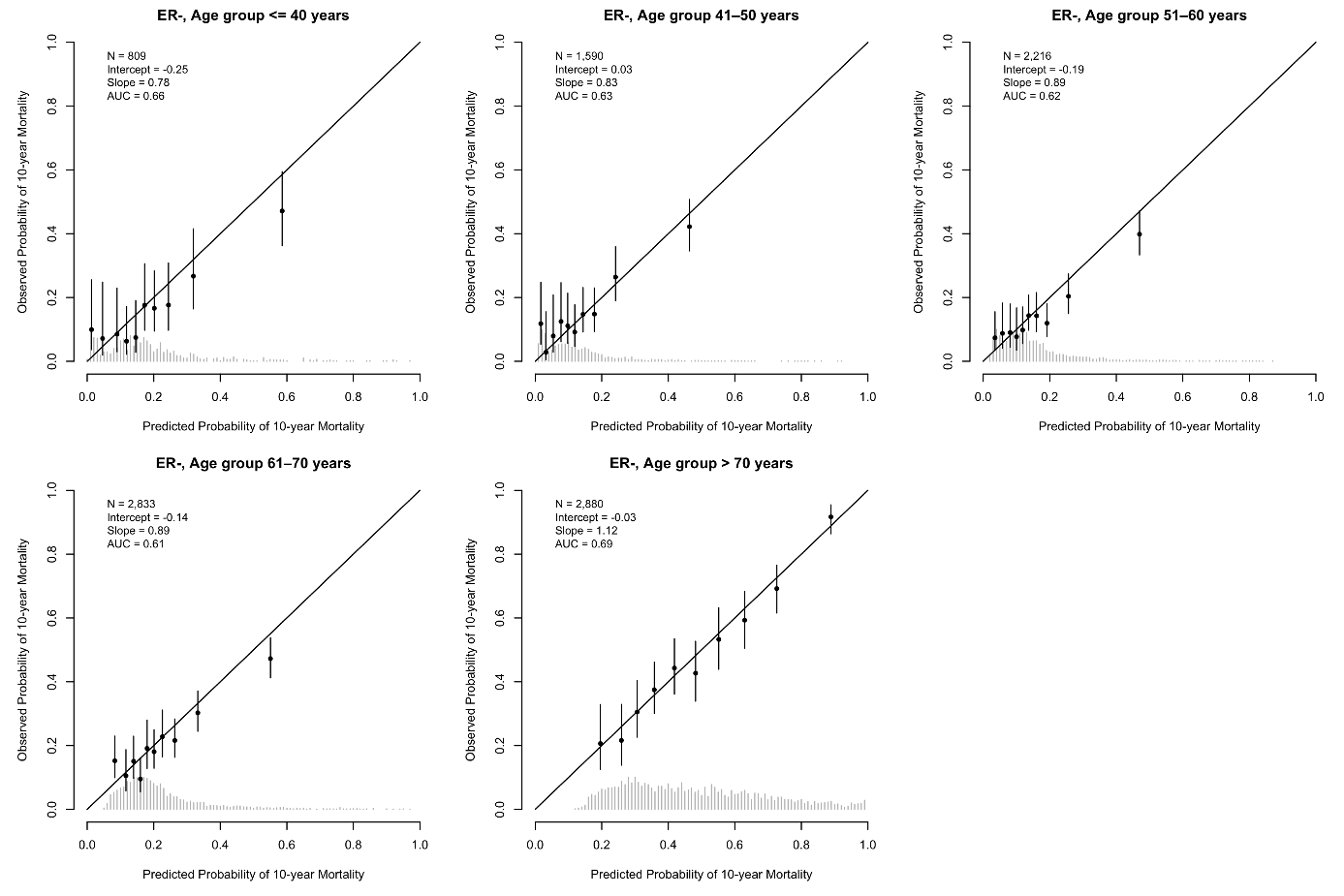

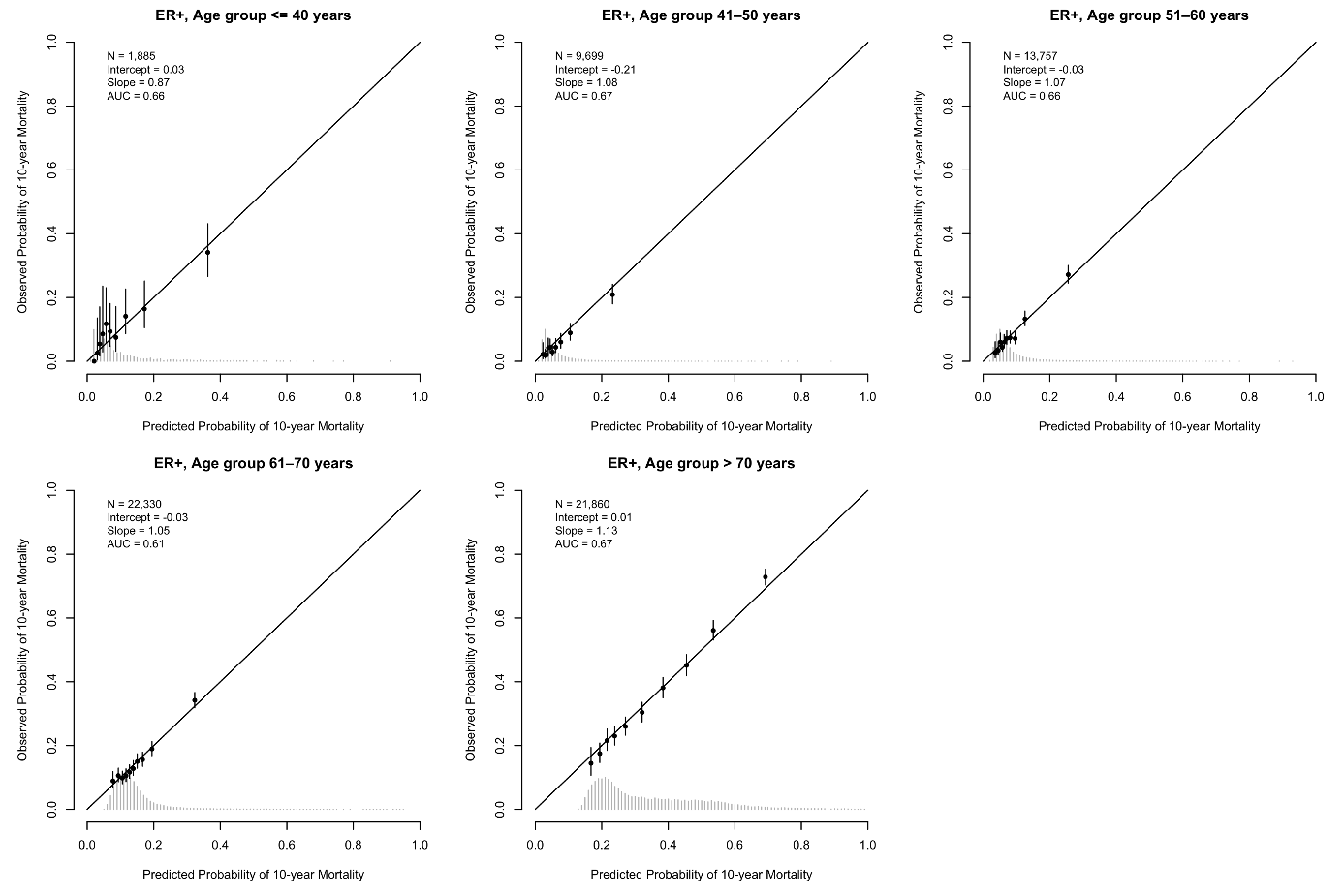


Supplementary Figure7A: Calibration plots stratified by age group and triple negative receptor status, for 10-year overall mortality in Dutch breast cancer patients using PREDICT Breast 3.1.


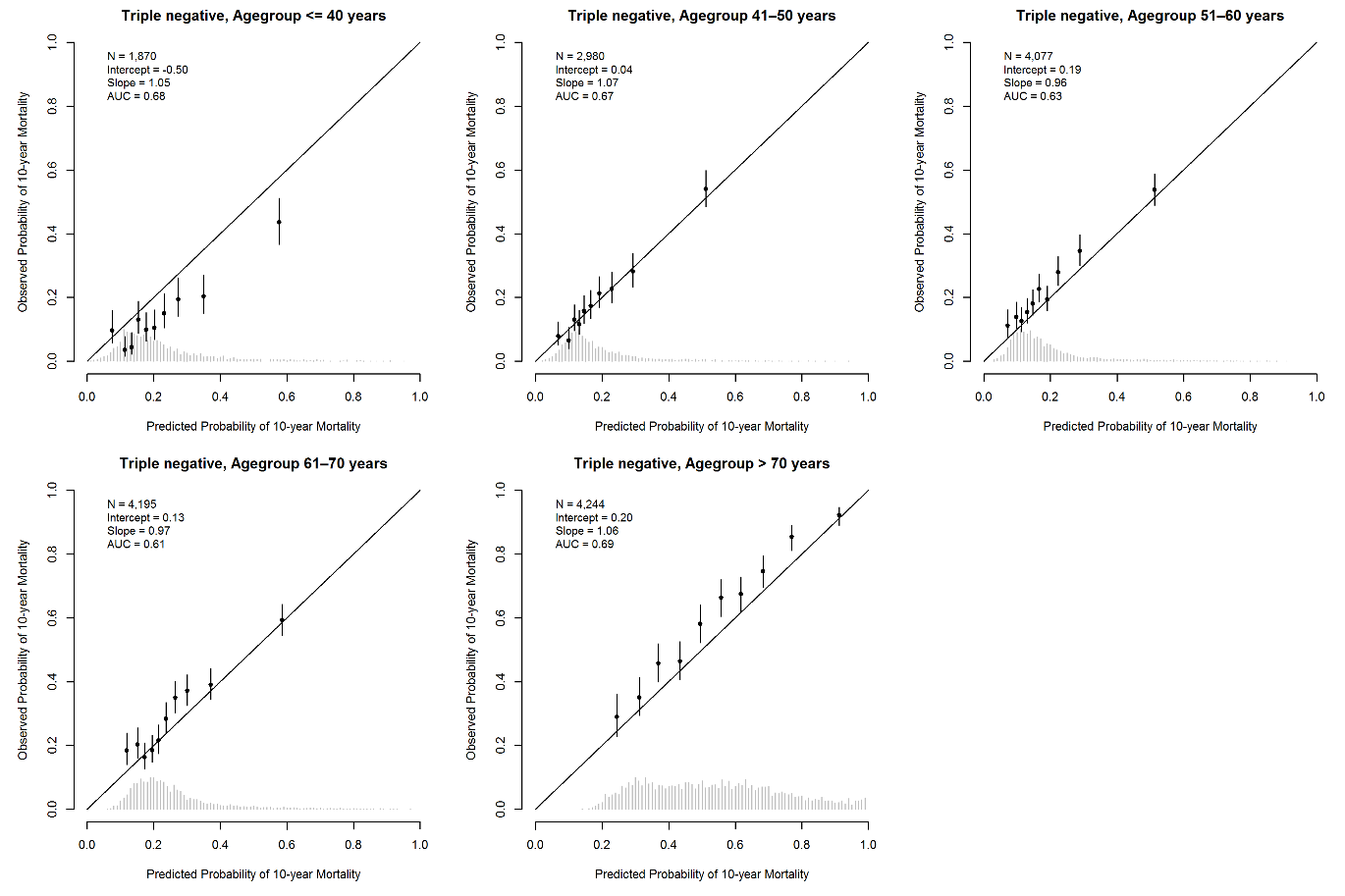


Supplementary Figure 7B: Calibration plots stratified by age group and triple negative receptor status, for 10-year overall mortality in Swedish breast cancer patients using PREDICT Breast 3.1.


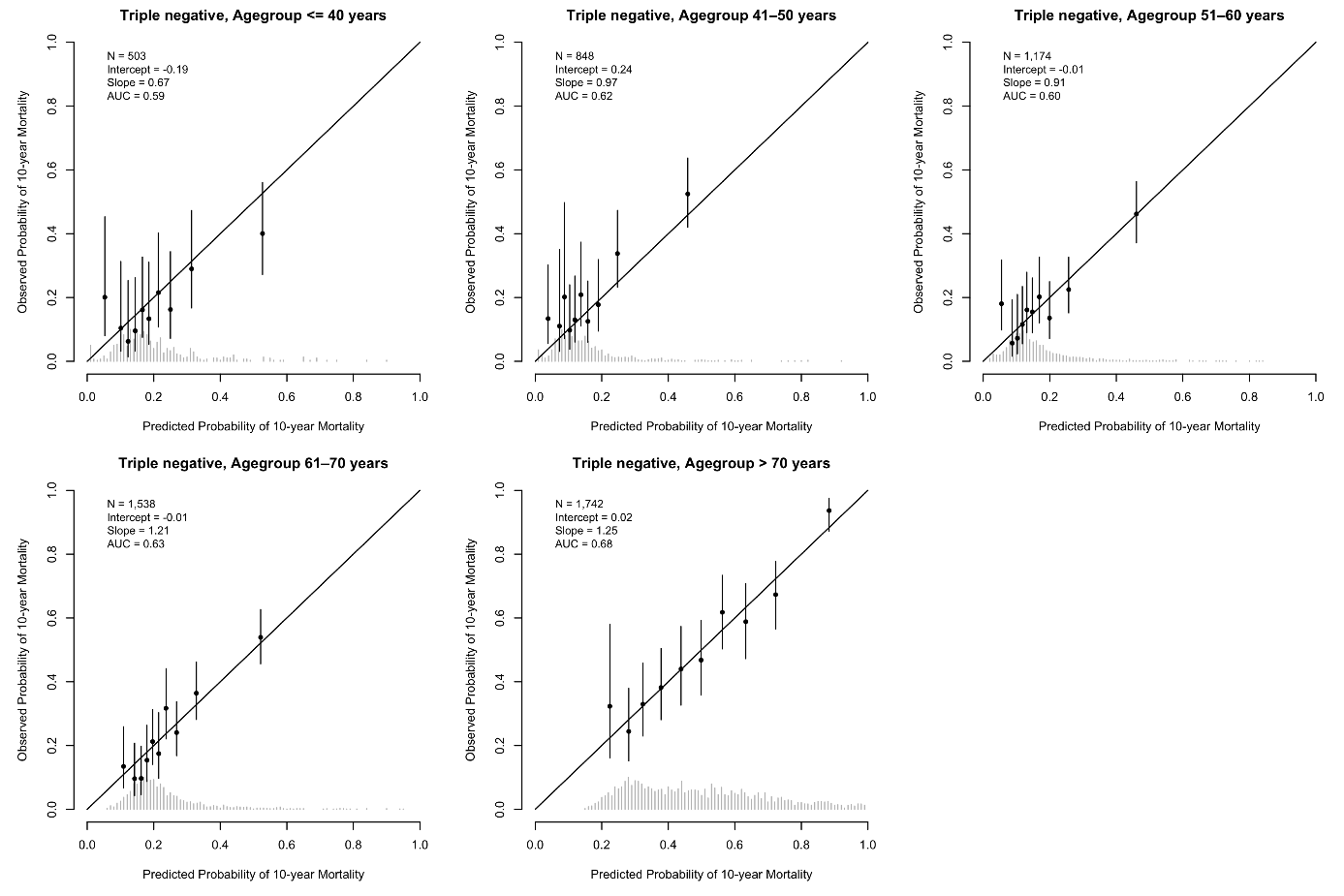


Supplementary figure 8: Calibration plots for patients treated with neoadjuvant chemotherapy from the Dutch (left) and Swedish (right) populations, showing 10-year overall mortality predictions using PREDICT Breast 3.1.


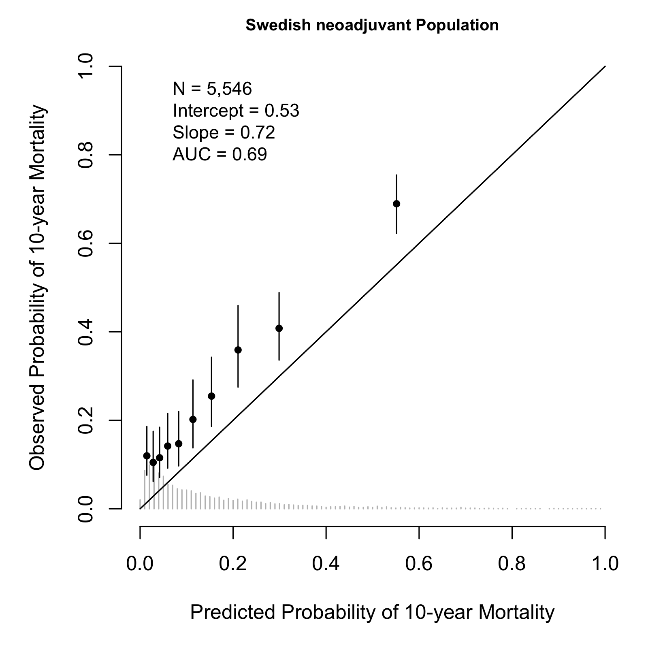

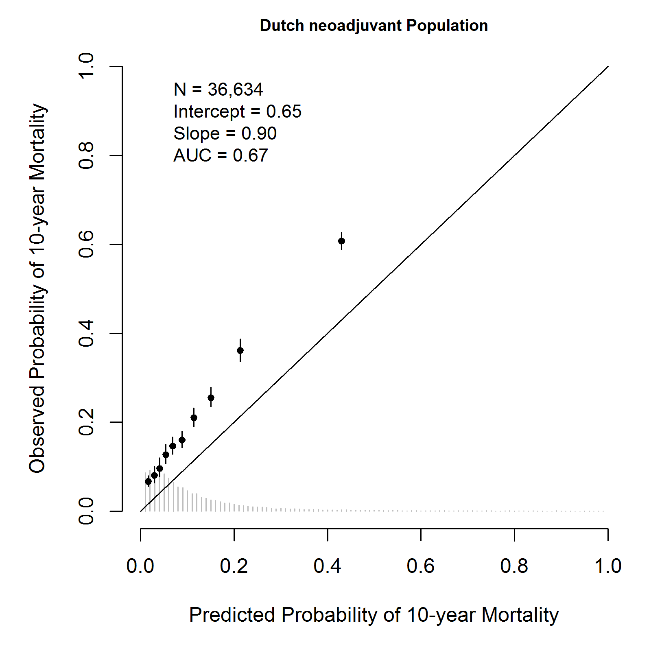


| **Section/Topic** | **Item** | **Checklist Item** | **Page** |
| --- | --- | --- | --- |
| **Title and abstract** | | | |
| Title | 1 | Identify the study as developing and/or validating a multivariable prediction model, the target population, and the outcome to be predicted. | 1 |
| Abstract | 2 | Provide a summary of objectives, study design, setting, participants, sample size, predictors, outcome, statistical analysis, results, and conclusions. | 2 |
| **Introduction** | | | |
| Background and objectives | 3a | Explain the medical context (including whether diagnostic or prognostic) and rationale for developing or validating the multivariable prediction model, including references to existing models. | 3 |
|  | 3b | Specify the objectives, including whether the study describes the development or validation of the model or both. | 3 |
| **Methods** | | | |
| Source of data | 4a | Describe the study design or source of data (e.g., randomized trial, cohort, or registry data), separately for the development and validation data sets, if applicable. | 4 |
|  | 4b | Specify the key study dates, including start of accrual; end of accrual; and, if applicable, end of follow-up. | 4, Supplementary Figures 1A,B,C |
| Participants | 5a | Specify key elements of the study setting (e.g., primary care, secondary care, general population) including number and location of centres. | 4 |
|  | 5b | Describe eligibility criteria for participants. | 4 |
|  | 5c | Give details of treatments received, if relevant. | 4,5 |
| Outcome | 6a | Clearly define the outcome that is predicted by the prediction model, including how and when assessed. | 5 |
|  | 6b | Report any actions to blind assessment of the outcome to be predicted. | - |
| Predictors | 7a | Clearly define all predictors used in developing or validating the multivariable prediction model, including how and when they were measured. | 4,5, Supplementary table 1 |
|  | 7b | Report any actions to blind assessment of predictors for the outcome and other predictors. | - |
| Sample size | 8 | Explain how the study size was arrived at. | 4 |
| Missing data | 9 | Describe how missing data were handled (e.g., complete-case analysis, single imputation, multiple imputation) with details of any imputation method. | 4,5, Supplementary table 1 |
| Statistical analysis methods | 10c | For validation, describe how the predictions were calculated. | 5,6 |
|  | 10d | Specify all measures used to assess model performance and, if relevant, to compare multiple models. | 5,6 |
|  | 10e | Describe any model updating (e.g., recalibration) arising from the validation, if done. | - |
| Risk groups | 11 | Provide details on how risk groups were created, if done. | 4,5 |
| Development vs. validation | 12 | For validation, identify any differences from the development data in setting, eligibility criteria, outcome, and predictors. | 4,5 |
| **Results** | | | |
| Participants | 13a | Describe the flow of participants through the study, including the number of participants with and without the outcome and, if applicable, a summary of the follow-up time. A diagram may be helpful. | Table 1 |
|  | 13b | Describe the characteristics of the participants (basic demographics, clinical features, available predictors), including the number of participants with missing data for predictors and outcome. | Table 1 |
|  | 13c | For validation, show a comparison with the development data of the distribution of important variables (demographics, predictors and outcome). | 16 |
| Model performance | 16 | Report performance measures (with CIs) for the prediction model. | 7-13, Table S3 |
| Model-updating | 17 | If done, report the results from any model updating (i.e., model specification, model performance). | - |
| **Discussion** | | | |
| Limitations | 18 | Discuss any limitations of the study (such as nonrepresentative sample, few events per predictor, missing data). | 15,16 |
| Interpretation | 19a | For validation, discuss the results with reference to performance in the development data, and any other validation data. | 14,15 |
|  | 19b | Give an overall interpretation of the results, considering objectives, limitations, results from similar studies, and other relevant evidence. | 14 t/m 16 |
| Implications | 20 | Discuss the potential clinical use of the model and implications for future research. | 14 t/m 16 |
| **Other information** | | | |
| Supplementary information | 21 | Provide information about the availability of supplementary resources, such as study protocol, Web calculator, and data sets. | 16 + supplementals |
| Funding | 22 | Give the source of funding and the role of the funders for the present study. | 16 |

Table S2: TRIPOD checklist prediction model validation.

|  | **AUC (CI) - Netherlands** | **AUC (CI) - Sweden** |
| --- | --- | --- |
| **40 years or younger** | 0.67 (0.65-0.69) | 0.66 (0.61-70) |
| **41–50 years** | 0.68 (0.67-0.69) | 0.68 (0.66-0.71) |
| **51-60 years** | 0.64 (0.63-0.65) | 0.66 (0.64-0.68) |
| **61-70 years** | 0.60 (0.59-0.60) | 0.61 (0.60-0.63) |
| **70 years or older** | 0.68 (0.68-0.69) | 0.67 (0.66-0.68) |

Table S3: Area under the receiver operating characteristic curve (AUC) at 10 years for the different age groups corresponding to Figures 3 and 4 in the main manuscript, including 95% confidence intervals (CIs).
